# DFT-assisted low-dimensional carbon-based electrocatalysts design and mechanism study: a review

**DOI:** 10.3389/fchem.2023.1286257

**Published:** 2023-10-17

**Authors:** Yun Han, Hongzhe Xu, Qin Li, Aijun Du, Xuecheng Yan

**Affiliations:** ^1^ Queensland Micro- and Nanotechnology Centre, Griffith University, Nathan Campus, Brisbane, QLD, Australia; ^2^ School of Engineering and Built Environment, Griffith University, Nathan Campus, Brisbane, QLD, Australia; ^3^ School of Chemistry and Physics and Centre for Materials Science, Queensland University of Technology, Gardens Point Campus, Brisbane, QLD, Australia

**Keywords:** density functional theory, descriptor, carbon-based materials, electrocatalysis, molecular dynamics

## Abstract

Low-dimensional carbon-based (LDC) materials have attracted extensive research attention in electrocatalysis because of their unique advantages such as structural diversity, low cost, and chemical tolerance. They have been widely used in a broad range of electrochemical reactions to relieve environmental pollution and energy crisis. Typical examples include hydrogen evolution reaction (HER), oxygen evolution reaction (OER), oxygen reduction reaction (ORR), carbon dioxide reduction reaction (CO_2_RR), and nitrogen reduction reaction (NRR). Traditional “trial and error” strategies greatly slowed down the rational design of electrocatalysts for these important applications. Recent studies show that the combination of density functional theory (DFT) calculations and experimental research is capable of accurately predicting the structures of electrocatalysts, thus revealing the catalytic mechanisms. Herein, current well-recognized collaboration methods of theory and practice are reviewed. The commonly used calculation methods and the basic functionals are briefly summarized. Special attention is paid to descriptors that are widely accepted as a bridge linking the structure and activity and the breakthroughs for high-volume accurate prediction of electrocatalysts. Importantly, correlated multiple descriptors are used to systematically describe the complicated interfacial electrocatalytic processes of LDC catalysts. Furthermore, machine learning and high-throughput simulations are crucial in assisting the discovery of new multiple descriptors and reaction mechanisms. This review will guide the further development of LDC electrocatalysts for extended applications from the aspect of DFT computations.

## 1 Introduction

**FIGURE 1:**
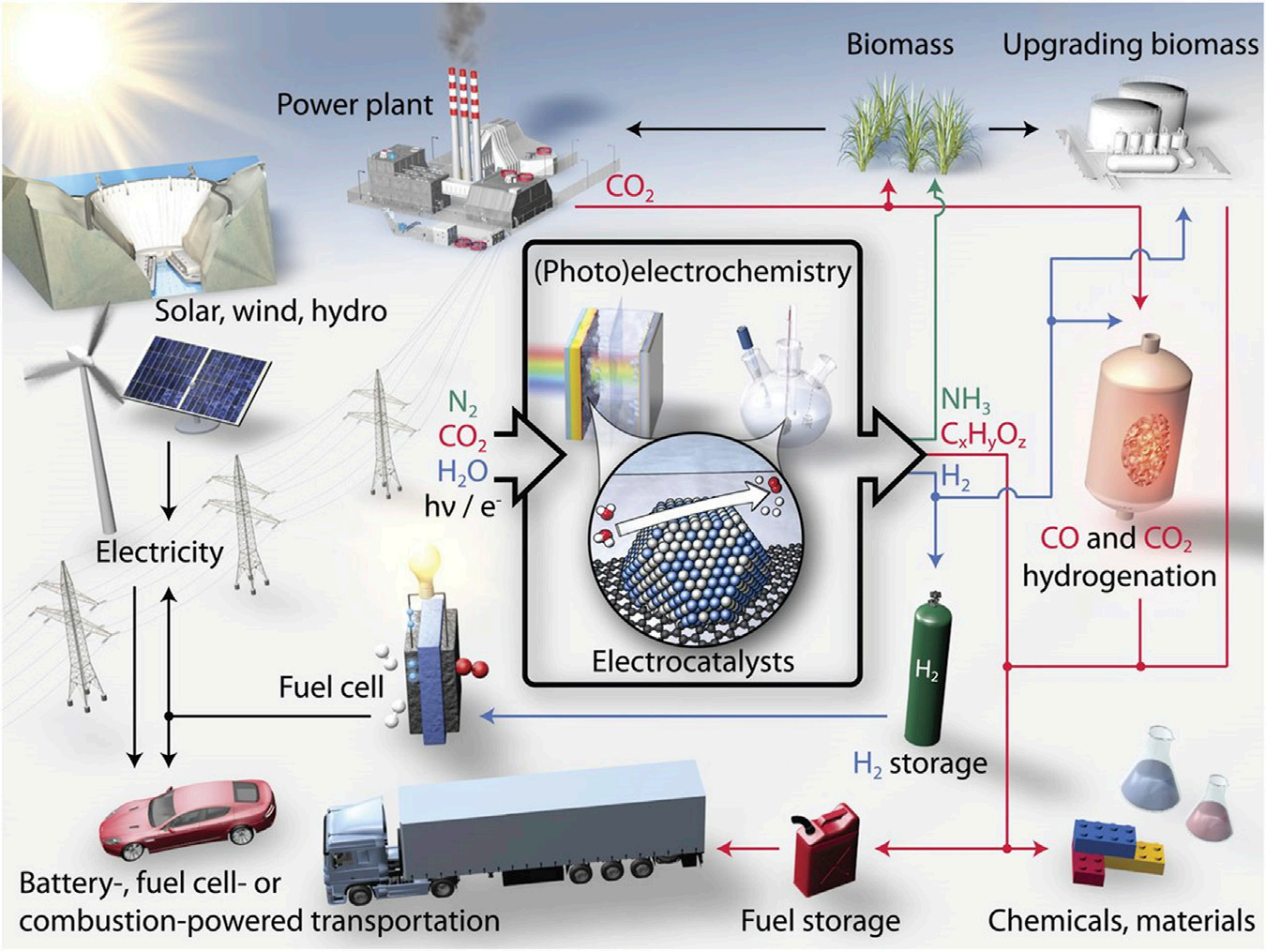
Schematic of a sustainable energy landscape based on electrocatalysis. Reproduced with permission (Seh et al., 2017). Copyright 2017, American Association for the Advancement of Science.

Environmental pollution and energy crisis are the two main critical issues of modern society caused by the excessive use of fossil fuels. Acid rain, haze, and greenhouse effects have disastrously affected the normal life of human beings (Turner John, 2004; Hubert and Nenad, 2009; Dai et al., 2015). On the one hand, great efforts have been devoted to the investigation and utilization of renewable clean energy and the efficient conversion between electrical and chemical energy, i.e., electrocatalytic hydrolysis and fuel cells (Chu and Majumdar, 2012; Abas et al., 2015). Greenhouse gas recycling, on the other hand, is also regarded as one of the most promising techniques to reduce air pollution, for example, the reduction of CO_2_. Therefore, effective electrochemical reactions, such as hydrogen evolution reaction (HER) and oxygen evolution reaction (OER) for water splitting, oxygen reduction reaction (ORR) for fuel cell, and carbon-dioxide reduction reaction (CO_2_RR) are highly expected to solve the above issues (Figure 1). (Karunadasa et al., 2010; Seh et al., 2017) However, the performance of these electrochemical reactions is severely hampered by their sluggish kinetics, which can be significantly improved with the introduction of efficient electrode catalysts (Liang et al., 2013; Zou and Zhang, 2015).

Noble metal-based materials such as platinum (Pt) and ruthenium (Ru) are widely used as benchmark catalysts. However, the scarcity and high cost impede their industrial mass production. Therefore, non-noble metals and even metal-free electrocatalysts have been intensively studied and remarkable progress has been reached (Yan X et al., 2016; Sui et al., 2017; Zhang L et al., 2018; Liu et al., 2019; Han et al., 2023a; He et al., 2023). Up to now, low-dimensional carbon materials, such as 2D graphene nanosheets (G), 1D carbon nanotubes (CNT), and 0D fullerenes, have been widely used for fabricating a series of non-noble metals and even metal-free catalysts for energy conversion. This is because of their unique properties such as tunable molecular structures, spatial confinement and surface effect, abundance and excellent oxidation, and corrosion resistance. Various defects and heteroatoms are embedded into carbon substrates, such as transition metals, nitrogen, and boron, which can greatly improve their catalytic efficiency (Kroto et al., 1985; Chen et al., 2002; Novoselov et al., 2004; Hsieh et al., 2009; Chen et al., 2013; Zhuo et al., 2013; Jia et al., 2016; Yan Y et al., 2016; He et al., 2017; Guo et al., 2018; Mao et al., 2019a).

It is difficult to reveal the catalytic mechanism and actual active sites of electrocatalysts only via experimental study. For instance, previous studies show that pyridinic nitrogen is responsible for the ORR in N-doped carbon-based metal-free catalysts (Wu et al., 2015; Liu and Dai, 2016), while other studies suggest that the active sites are graphitic nitrogen (Liu et al., 2010; Lin et al., 2013). Therefore, the current quest is to develop appropriate methodologies to provide a comprehensive understanding of the catalyst structures at electronic levels, which will promote the comprehension of the reaction mechanisms and guide future experimental studies (Bora et al., 2019). Nowadays, based on the first principles, the modern density functional theory (DFT) calculation has become an irreplaceable modeling toolkit for scientists in a variety of research areas. Two main strategies of DFT calculation-assisted design of electrocatalysts have been established by theoretical and experimental chemists. The first one is dominated by theoretical calculations, dedicated to achieving a rational design of high-performance catalysts. It is crucial to identify the most important parameters to reveal the relationships between structures and performance, which are the so-called descriptors and can considerably boost the traditional trial-and-error approach. Machine-learning and high-throughput calculations have also been developed to efficiently screen active sites and descriptors for the targeted catalysts. The second one is dominated by experimental testing and characterizations, assisted by calculating the change during the catalysis process such as step-energy, molecular and electronic structure, and electron transition that can reveal the reaction mechanism accurately. In this review, we will mainly summarize the history and concepts of modern DFT, the heterogeneous electrocatalytic surface-related descriptors, the combination of experiment and calculation, and recent achievements of low-dimensional carbon-based electrocatalysts for energy storage and conversion applications.

## 2 Overview of DFT

Quantum chemistry is mainly based on quantum mechanics principles, and the main goal of all first-principle calculations is to obtain the electronic wave function *ψ* that characterizes the state of the system, for which the Schrödinger equation must be solved. Theoretical computing has received increasing attention from the chemistry community as computer technology has progressed. However, the traditional Wave Function Theory (WFT) method has two fatal shortcomings. First, the wave function *ψ* of a system with N electrons will contain 3N coordinate variables. Therefore, a 3N-dimensional wave function image, which is difficult to describe visually, will be a stumbling block in solving the Schrödinger equation. Second, the Schrödinger equation for a multi-electron system is too computationally intensive to be calculated accurately. Modern DFT has become a viable option to solve these drawbacks.

### 2.1 History of modern DFT

The homogeneous electron-gas model, commonly known as the Thomas–Fermi model, was proposed by Thomas and Fermi in 1927, and it has established a firm foundation for DFT. The ground state energy of the electron system is directly represented in terms of electron density instead of the wave function, which drastically reduces the freedom degree of the system (Thomas, 1927). In 1964, Hohenberg and Kohn proposed an inhomogeneous electron-gas model based on the Thomas–Fermi model and proved that two theorems served as the fundamentals of modern DFT (Hohenberg and Kohn, 1964). The first theorem stated that the nuclear potential energy $V\left(r\right)$ of all electrons in a system that has *n* interacting electrons is a unique function of the electronic density $\rho \left(r\right)$ . This could be presented as the Hohenberg-Kohn (HK) equation as shown in Eq. (2.1).

$$E_{V}\left[\rho \right]=\rho \left(r\right)V\left(r\right)dr+F_{HK}\left[\rho \right]$$ (2.1)

Here, $F_{HK}\left[\rho \right]=T\left[\rho \right]+E_{ee}\left[\rho \right]$ , where $T\left[\rho \right]$ is the sum of electronic kinetic energy and $E_{ee}\left[\rho \right]$ is electron–electron repulsion. Clearly, $F_{HK}\left[\rho \right]$ is independent of the external potential field. However, the HK equation cannot be directly employed for the calculation of the total energy of the system due to the unknown specific form of $F_{HK}\left[\rho \right]$ . The second theory proposed a density minimum principle, stating that the ground state energy of any trial electron density $\rho \left(r\right)$ cannot be lower than the true ground system. Hence, it can be inferred that as the $\rho \left(r\right)$ approaches the true electron density, the calculated system energy becomes closer to the ground-state energy of the system. Then the Euler-Lagrange equation can be obtained as shown in Eq. (2.2).

$$\mu =\frac{\delta E_{V}\left[\rho \right]}{\delta \rho \left(r\right)}=V_{cxt}\left(r\right)+\frac{\delta F_{HK}\left[\rho \right]}{\delta \rho \left(r\right)}$$ (2.2)

Where, $V_{cxt}\left(r\right)$ represents an external potential. Therefore, if $F_{HK}\left[\rho \right]$ is confirmed, $\rho \left(r\right)$ and $E_{V}\left[\rho \right]$ can be solved by Eq. (2.2). However, the exact expression for $F_{HK}\left[\rho \right]$ remains an unsolved challenge until now. In 1965, building upon the Thomas–Fermi model’s shortcomings and drawing inspiration from the Hartree-Fock method, Kohn and Sham revealed a brand-new approach to derive the $F_{HK}\left[\rho \right]$ approximately. $F_{HK}\left[\rho \right]$ can be divided into three parts and can be presented: (Kohn and Sham, 1965).

$$F_{HK}\left[\rho \right]=T_{s}\left[\rho \right]+J\left[\rho \right]+E_{xc}\left[\rho \right]$$ (2.3)

Where, $T_{s}\left[\rho \right]$ and $J\left[\rho \right]$ are the kinetic energy and coulombic correlation energy of independent electrons, respectively. $E_{xc}\left[\rho \right]$ is a functional with a small magnitude called exchange–correlation (XC) energy that can be approximated, and it can be presented as:

$$E_{xc}\left[\rho \right]=T\left[\rho \right]-T_{s}\left[\rho \right]+V_{ee}\left[\rho \right]-J\left[\rho \right]$$ (2.4)

Due to $T_{s}\left[\rho \right]$ and $J\left[\rho \right]$ comprising the primary components of Fock exchange energy $F_{HK}\left[\rho \right]$ , the remaining $E_{xc}\left[\rho \right]$ is a functional with relatively small values. Even when subjected to approximate treatment, the errors associated with this part are not particularly large. By variation, we can get the famous Kohn–Sham (KS) equation:

$${\hat{H}}_{KS}\emptyset_{i}\equiv \left[-\frac{1}{2}\nabla^{2}+V\left(r\right)+\int \rho \left(r^{\prime }\right)/\left|r-r^{\prime }\right|dr^{\prime }+V_{xc}\left(r\right)\right]\emptyset_{i}\left(r\right)=\varepsilon_{i}\emptyset_{i}\left(r\right)$$ (2.5)

And XC potential can be defined by:

$$V_{xc}\left(r\right)=\delta E_{xc}\left[\rho \right]/\delta \rho \left(r\right)$$ (2.6)

Overall, there is no approximation and the Kohn–Sham equation is accurate. However, the $E_{xc}\left[\rho \right]$ component of the Kohn–Sham equation still lacks a rigorous expression form, making it impossible to use in practical calculations. Instead, several types of approximate XC generalized functionals must be built to gradually approximate the real system. In simple terms, when solving the KS equations, we need to start with an initial guess for the electron density, use it to solve the KS orbitals, and then calculate the electron density distribution function with the obtained KS orbitals. This distribution function is then used in the KS equations for further iterations, repeating this process until the electron density distribution converges. This iterative approach, which progressively refines the solution through approximations, is the main source of errors in DFT calculations.

### 2.2 Exchange–correlation (XC) functional

**FIGURE 2:**
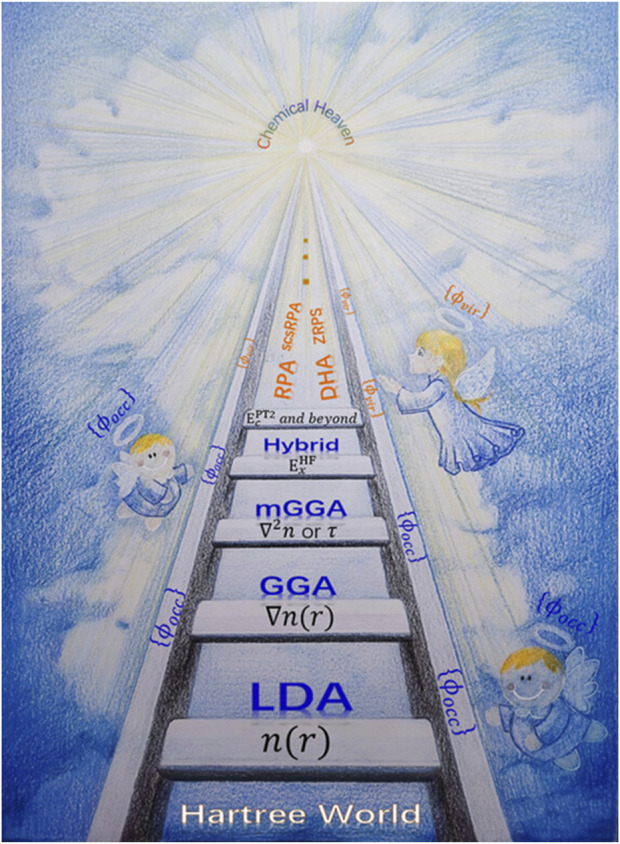
Illustration of Jacob’s Ladder of DFT. Reproduced with permission. (Zhang et al., 2020). Copyright 2020, John Wiley and Sons.

Typical approximations such as local density approximation (LDA), generalized gradient approximation (GGA), meta-GGA, and hybrid functional are shown in the well-known Jacob’s ladder from Hartree world to Chemical accuracy heaven (Figure 2). (Mattsson, 2002; Tao et al., 2003; Zhang and Xu, 2020)

The approximation for LDA is that the charge density changes slowly with position and behaves similarly to the local heterogeneous electron cloud, so the XC energy can be presented as:

$$E_{XC}^{LDA}\left[n\right]=\int drn\left(r\right)\varepsilon_{XC}\left(n\right)$$ (2.7)

The LDA method only considers local charge density, which is accurate for a uniform electron gas model but is clearly not applicable to real systems where the electron density is not perfectly uniform. Therefore, this method provides a rough approximation when computing the real systems and tends to overestimate binding energies.

To improve the accuracy of the LDA method, the GGA method describes electron density with the assistance of density gradient and can be presented as:

$$E_{XC}^{GGA}\propto \int drf\left(n,\nabla n\right)$$ (2.8)

BLYP (Becke, 1988; Lee et al., 1988), PW91 (Perdew et al., 1992; White and Bird, 1994), and PBE (Perdew et al., 1996) are the commonly used approximated density functionals of the GGA method. The Becke exchange functional is a gradient-corrected exchange functional that considers the gradient of the electron density. The Lee-Yang-Parr (LYP) correlation functional incorporates the correlation energy of electrons. By combining these two components, BLYP aims to provide a more accurate description of electron-electron interactions and correlation effects in molecular systems. PW91 combines the Perdew-Wang exchange functional with the Perdew-Wang correlation functional. The exchange functional accounts for the exchange energy of electrons, while the correlation functional deals with electron-electron correlation effects. The PBE functional captures both the exchange and correlation effects in the electron-electron interaction. It has been found to perform well for a wide range of systems, including molecules, solids, and surfaces, making it a popular option in DFT calculations.

Based on the GGA method, meta-GGAs, such as TPSS, TPSSh, and M06-L (Zhao et al., 2006; Zhao and Truhlar, 2006; Zhao and Truhlar, 2008), introduced kinetic energy density variables but with better accuracy accompanied by a significant increase in computational cost.

By partially combining accurate XC functional in the DFT, the hybrid functional could improve the calculation accuracy. The most popular one is the B3LYP functional (Stephens et al., 1994), which can be described as:

$$E_{XC}^{B3LYP}=A∙E_{X}^{LDA}+\left(1-A\right)E_{X}^{HF}+B∙∆E_{X}^{Beck}+C∙E_{C}^{LYP}+\left(1-C\right)E_{C}^{VWN}$$ (2.9)

Where A = 0.8, B = 0.72, and C = 0.81. $E_{X}^{LDA}$ is the LDA exchange functional, $E_{X}^{HF}$ is the Hartree–Fock exact exchange functional, $∆E_{X}^{Beck}$ is the Becke exchange functional, $E_{C}^{LYP}$ is the LYP correlation functional, and $E_{C}^{VWN}$ is the VWN local spin density approximation to the correlation functional.

Calculations involving periodic systems frequently employ the HSE functional (Heyd and Scuseria, 2004), which is represented by the following equation:

$$E_{XC}^{HSE}=A∙E_{X}^{HF,SR}\left(\omega \right)+\left(1-A\right)E_{X}^{PBE,SR}\left(\omega \right)+E_{X}^{PBE,LR}\left(\omega \right)+E_{C}^{PBE}$$ (2.10)

Where A is the mixing parameter, and *ω* is an adjustable parameter controlling the short range of the interaction. The standard values of *A* = 0.25 and *ω* = 0.2 of HSE06 (Heyd et al., 2003) have been shown to give good results for most systems. $E_{X}^{HF,SR}\left(\omega \right)$ is the short-range Hartree-Fock exact exchange functional, $E_{X}^{PBE,SR}\left(\omega \right)$ and $E_{X}^{PBE,LR}\left(\omega \right)$ are the short- and long-range components of the PBE exchange functional, and $E_{C}^{PBE}$ is the PBE correlation functional.

### 2.3 Periodic system

To deal with periodic systems, supercell models (primitive cells in three dimensions (X, Y, and Z) with periodic repetition) are frequently used. Vacuum space greater than 10 Å is added in the *Z*-direction to eliminate the interaction. In condensed matter physics, Bloch’s theorem states that solutions to the Schrödinger equation in a periodic potential take the form of a plane wave modulated by a periodic function and can be written as:

$$\Psi \left(r\right)=u_{k}\left(r\right)e^{ikr}$$ (2.11)

Where *r* is the position, *Ψ* is the wave function, and *k* is the wave vector. Based on the Bloch theorem, the electronic band structure is involved in investigating surface science, and electronic states at numerous *k* points should be calculated. In practical calculations, however, a finite number of *k* points is used; the higher the density of the *k* points, the lower the inaccuracy will be. In general, the number of *k* points for metal systems is higher than that of the oxide systems.

For periodic systems, the electron wave function can be expanded with numerous plane wave functions. However, only plane-wave basis sets with kinetic energy lower than the cut-off energy are considered in practical calculations, which would result in a systematic error (Kresse and Furthmüller, 1996a; Kresse and Furthmüller, 1996b). Therefore, a pseudopotential that can replace the true atomic potential of the nucleus and inner electrons is introduced to reduce systematic error. Currently, the most widely used plane-wave methods are the ultra-soft pseudopotential (US-PP) plane wave method and the projector augmented wave (PAW) method (Kresse and Joubert, 1999).

### 2.4 DFT + U scheme

DFT calculations cannot adequately describe systems with physical properties that are controlled by many body electronic interactions (correlated systems) because it is typically challenging to model the dependence of the XC functional on electronic charge density. The over-delocalize valence electrons and over-stabilize metallic ground states are considered to be the main problems of DFT to describe correlated systems. Therefore, the DFT + U approach has been developed to enhance the description of the ground state of correlated systems (Dudarev et al., 1998). The key advantage of the DFT + U method is that it is within the realm of DFT, making it easy to implement in the existing DFT codes. Besides, its computational cost is only slightly higher than that of normal DFT computations. The local and semi-local density functionals that allow LDA + U and GGA + U computational operations can be enhanced with this ‘U’ correction. The primary function of the ‘U’ correction is to add an additional Hubbard-like term to the strong on-site Coulomb interaction of localized electrons. The on-site Coulomb term U and the site exchange term J are the two parameters that represent the strength of the on-site interactions for the purpose of practical DFT + U implementation in computational chemistry. Parameters ‘U and J’ are typically derived semi-empirically although *ab initio* computations can yield them (Tolba et al., 2018).

$$E\left(DFT+U\right)=E\left(DFT\right)+U_{eff}/2\sum_{\sigma }Tr\left[\rho^{\sigma }-\rho^{\sigma }\rho^{\sigma }\right]$$ (2.12)

It has become a standard to use the parameter: $U_{eff}=U-J$ in place of the interaction U in the simplified LDA + U form. $U_{eff}$ is frequently utilized because the ‘J’ has been shown to be essential for describing the electronic structure of specific classes of materials, typically those subject to high spin-orbit coupling.

### 2.5 Molecular dynamics simulation

Molecular dynamics (MD) simulation, which is based on Newton’s laws of motion, can simulate the trajectory of each atom in a system at a certain temperature, thus the system’s dynamic properties can be calculated. However, the standard DFT can only be used to calculate a system’s static properties at 0 K. Briefly, for a given configuration and initial velocity of each atom, the MD simulation can be divided into three steps. Firstly, the forces acting on each atom in the system are calculated to determine the system’s acceleration. Secondly, the configuration of the system after Δ*t* time can be obtained according to Newton’s three laws. Thirdly, the calculation of the forces in the first step is continued based on this configuration, and the results of the system evolving with time can be finally obtained by repeated recurrence. MD simulation can be further classified into two types: Classic MD simulation and *ab initio* MD (AIMD) simulation, depending on how the forces are calculated in the first step. Classic MD simulation builds the potential energy function from the empirical force field and calculates the force at each step using the gradient of the potential energy function. AIMD simulation, on the other hand, can be used to accurately calculate the force at each step by the *ab initio* quantum chemical method (Nosé, 1984; Hoover, 1985; Kresse and Hafner, 1993).

Unfortunately, AIMD is constrained by its suitability for small simulation systems and limited simulation durations. The utilization of force fields, however, can expedite the computation process. Nevertheless, for an extended period, there existed a gap between computationally demanding electronic structure-based DFT calculations and the more efficient yet less precise empirical potentials or force fields founded on physical approximations and intuition (Kocer et al., 2022). This situation witnessed substantial improvement with the emergence of machine learning potentials (MLPs) in 1995 (Blank et al., 1995). Modern MLPs have the capacity to discern the shape of multidimensional potential energy surfaces from high-level DFT calculations and subsequently incorporate the derived analytical atomic interactions (force fields) into large-scale simulations, such as MD, with negligible accuracy compromise (Handley and Popelier, 2010; Friederich et al., 2021; Unke et al., 2021). Over the last two decades, numerous types of MLPs have been introduced, including neural network potentials (NNPs) (Lorenz et al., 2004; Behler and Parrinello, 2007), Gaussian approximation potentials (GAPs) (Bartók et al., 2010), and gradient domain machine learning (GDML) (Chmiela et al., 2019). Among them, NNPs exhibit a formidable combination of neural network expressive capabilities and the availability of extensive datasets like the QM9 dataset (Ruddigkeit et al., 2012), Material Project (Jain et al., 2013), and Open Catalyst 2020 (Chanussot et al., 2021), rendering them exceptionally well-suited for expediting high-accuracy MD calculations (Takamoto et al., 2022). Additionally, beyond reliance on existing databases, on-the-fly machine learning enables the construction of precise force fields from newly sampled data with minimal training overhead (Jinnouchi et al., 2019; Jinnouchi et al., 2020). In this adaptive process, during each step, a decision is made whether to perform an *ab initio* calculation and potentially incorporate the data into the force field or to employ the existing force field and skip the learning phase for that specific step.

Furthermore, Metadynamics is an atomistic simulation technique based on MD that operates within the same system to expedite the exploration of rare events and calculate the free energies of intricate molecular systems (Laio and Parrinello, 2002; Iannuzzi et al., 2003). This technique functions by iteratively ‘filling’ the system’s potential energy surface using a series of Gaussian functions centered along its trajectory, all guided by a carefully selected set of collective variables (CVs). This process compels the system to transition from one energy minimum to another (Bussi and Laio, 2020). Metadynamics provides several advantages in free energy calculations. Firstly, it accelerates the sampling of high-energy events within the studied system by progressively moving it from low-energy regions to high-energy areas via the inclusion of a sequence of repulsive potentials. Secondly, it facilitates the establishment of high-dimensional reaction pathways, enabling the computation of multidimensional free energy data, including two-dimensional free energy potential surfaces. Thirdly, because it gradually elevates the studied system’s energy from low to high regions, this approach excels in discovering optimal reaction pathways. Lastly, this method does not require a prior prediction of the reaction pathway within the studied system (Barducci et al., 2011). One limitation of Metadynamics lies in the difficulty of determining the optimal point for terminating the simulation. Without proper termination, the bias potential keeps increasing, possibly leading the system intoreaction regions that hold no interest in our study. The application of Well-Tempered Metadynamics effectively mitigates this limitation (Barducci et al., 2008).

### 2.6 Transition state theory

The transition state (TS), also known as the saddle point, is the highest energy point on the potential energy surface (PES) where the reactants (initial state, IS) generate products (final state, FS) along the minimum energy path. Within the transition state theory (TST), we assume that IS and TS are in quasi-equilibrium, and the main task is to find out the free energy barrier for the transition, which is the energy difference between IS and FS. Based on TST, the transition rate can also be calculated. The Dimer method (Henkelman and Jónsson, 1999) and the climbing image nudged elastic band (CINEB) approach (Henkelman et al., 2000) are the two most popular calculating techniques.

The dimer method can be used to find out the saddle points by two images of the system, which is the so-called ‘dimer’. Driven by the saddle point search algorithm, the dimer is moved uphill on the potential energy surface, away from the vicinity of the potential energy minimum of the IS and toward a saddle point. The dimer is rotated along the way to identify the potential energy mode with the lowest curvature at the dimer’s location. Since the Dimer technique only uses the first derivatives of the energy, the major benefit is not requiring the time-consuming computation of the minimal eigenvalue of the Hessian matrix. The Dimer technique also has the advantage of having less stringent initial configuration requirements.

The nudged elastic band (NEB) method is an efficient method for identifying the minimum energy path (MEP) between a given initial and the final state of a transition, which has also been widely used to estimate transition rates. A set of images between the IS and FS of the system are created, often on the order of 4–20. Then the adjacent images are contacted by a collection of spring interactions, which can form an elastic band and ensure the continuity of the path. The band is brought to the MEP by an optimization process that minimizes the force acting on the images. The drawback of the NEB method is that it requires the intermediates to be evenly spaced throughout the optimization process, and the predicted TS may be slightly deviated from the actual TS. The CINEB method was developed to address this issue. The force on the highest image is the full force resulting from the potential with the component along the elastic band inverted, showing that it will not be influenced by spring forces. As a result, the intermediates will gradually move to the higher energy direction and reach the actual TS.

### 2.7 Advanced simulation tools and methods

Currently, the computational hydrogen electrode (CHE) model is the most commonly employed and straightforward model for assessing catalytic activity (Peterson et al., 2010). This model uses half of the chemical potential of H_2_ in the gas phase at 0 V to represent the chemical potential of a proton-electron pair ( $G\left(\frac{1}{2}H_{2}\right)=G\left(H^{+}+e^{-}\right)$ ). When a potential U is applied to the catalytic system, the chemical potential can be adjusted as (Peterson et al., 2010):

$$G\left(\frac{1}{2}H_{2}\right)-eU=G\left(H^{+}+e^{-}\right)$$ (2.13)

Where *e* is the positive charge. Consequently, it becomes feasible to calculate the reaction-free energy (Δ*G*) of proton-coupled electron transfer (PCET) reactions, enabling a qualitative assessment of catalytic activity. Nonetheless, the CHE model falls short of representing the exact catalytic environment due to the neglection of sensitive parameters affecting catalytic performance, such as solvent, pH, and applied potential. To achieve a more precise simulation of electrochemical reactions, multiple approaches and tools have been developed and applied to consider the impact of the reaction environment.

#### 2.7.1 Solvent effect

Two primary approaches can be used to address the solvent effects: implicit and explicit solvation models. The implicit solvation model employs a polarizable medium, typically characterized by the dielectric constant (*ε*), to represent the solvent, while an electric field is constructed to depict the solvent’s charge distribution (Xu and Carter, 2019). Currently, two representative implicit solvation models are the solvation model based on solute electron density (SMD) (Marenich et al., 2009) and the conductor-like solution model (COSMO) (Klamt and Schüürmann, 1993). While the implicit solvation model permits a qualitative description of solvent effects at minimal computational cost, it falls short in describing specific interactions, such as hydrogen bonds (Ling et al., 2022).

On the other hand, the explicit solvation model precisely incorporates solvent molecules, atoms, and cations into the calculation systems, enabling direct observation of electrode/electrolyte and adsorbates/electrolyte interactions. When considering the most common water/electrode interfaces in electrocatalysis, two explicit solvation models are generally employed: icelike water bilayers and the electric double layer (Gross and Sakong, 2022). In the meantime, AIMD is typically employed to obtain reliable atomic configurations of the electrode/electrolyte interfaces, incurring significantly higher computational costs. Therefore, to strike a balance between computational accuracy and cost-effectiveness, the Quantum Mechanics/Molecular Mechanics (QMMM) method has been developed. With this approach, quantum mechanical (QM) computations are employed to simulate critical reaction components, such as adsorbates and electrode/electrolyte interfaces, while classical force fields or implicit solvation models are employed to handle the surrounding solvent environment. The primary challenge in the QMMM method is effectively partitioning the QM and MM fields to ensure an accurate representation of critical effects (Li et al., 2020).

#### 2.7.2 Applied electrode potential (U)

In experimental macroscopic systems, both reaction temperatures and the chemical potential of the electrolyte and electrode remain constant throughout the reaction. However, in most common DFT calculations, a limited number of atoms is typically treated within the canonical (NVT) or NPT ensemble and the total number of electrons in the simulated cells remains constant. Consequently, this leads to significant variations in the electrochemical potential (Fermi level) during a reaction. To address this problem and achieve a setup closer to the fluctuating particle numbers found in experimental conditions, the combination of the grand-canonical (GC) ensemble (VT*μ*) with DFT emerges as a natural choice for atomistic simulations (Melander et al., 2018). GC-DFT provides a method to investigate electrochemical microscopic systems in thermodynamic equilibrium, offering insights into phenomena characterized by long-time and length scales (Groß, 2021). During the fixed-potential grand canonical calculations, varying the total charge of the system can result in the fixed electrode potential (Fermi level) (Gao and Wang, 2021). Furthermore, by the fixed-potential method, we can easily calculate the potential-dependent reaction barriers and avoid the inaccurate assumption about the charge transfer coefficients in the constant-charge calculations. Currently, great developments have been reached in the field of methodology and models, such as continuum charging methods (Gauthier et al., 2019) and constant-potential hybrid-solvation dynamical model (CP-HS-DM) (Zhao and Liu, 2021), making complicated NEB and AIMD simulations stable and possible.

## 3 Theoretical guidance in catalyst design and mechanism study

Nowadays, a series of well-known carbon-based electrocatalysts have been fabricated through the combinations of various modification methods and substrate materials, such as heteroatom-doped graphene, defective graphene, Mxenes, heteroatom-doped carbon nanotubes/ribbons, and modified carbon dots (Yan et al., 2020; Zhou et al., 2020; Li et al., 2021; Ding et al., 2022; Song et al., 2022; Sun et al., 2022; Tang et al., 2022; Wu Q. et al., 2022; Wu Y. et al., 2022). However, the design of state-of-the-art electrocatalysts still relies on inefficient trial-and-error approaches, and the catalytic mechanism is still controversial and difficult to reveal only through experimental research. DFT calculations are thus utilized to address two issues: 1) predicting catalytic performance and guiding the synthesis of electrocatalysts and 2) investigating and corroborating the mechanism of electrocatalysis in conjunction with experimental study.

### 3.1 Application of theoretical guidance in designing electrocatalysts: descriptors

To predict the performance of electrocatalysts, computational scientists mainly focus on the most important microscopic properties of the catalysts that determine the macroscopic catalytic performance. Since the early 20th century, catalysts with high activity, according to the traditional Sabatier principle, should bind atoms and molecules with an ideal bond strength: not too weak to activate the reactants and not too strong to desorb the products. Therefore, a volcano-shape relationship between bond strength and catalytic activity has been proposed to represent the predictivity of many descriptors (Che, 2013). Due to the lack of quantitativeness and inability to undergo experimental validation, this principle is not very predictive. The “bond strength” between the relevant intermediates and the catalysts has been described during the past decades by the extraction of electronic and structural properties such as the descriptors.

#### 3.1.1 *d*-band center theory

**FIGURE 3:**
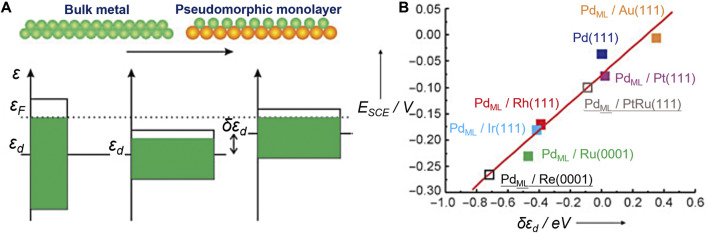
Illustration of the use of the *d*-band model. **(A)** The main origin of a shift in the *d*-band center *ε*_d_ is a change in the interatomic distances within an overlayer. **(B)** Electrochemically measured changes in the hydrogen adsorption energy (*E*_SCE_) for Pd overlayers on a number of metals are shown to scale well with the calculated shift of the *d*-band center (*δ*ε_d_). Reproduced with permission (Kibler et al., 2005). Copyright 2005, John Wiley and Sons.

The well-known *d*-band center theory proposed by Nørskov *et al.* has laid the foundation for a series of descriptors (Nørskov, 1991; Hammer and Norskov, 1995; Nilsson et al., 2005). It explains the interactions between atoms or molecules and the surface based on adsorbate orbitals and *d*-orbitals of atoms on the surface. The energy of the *d* states (the center of the *d* states) relative to the Fermi level is a good indicator of the bond strength. The higher the *d* states are in energy relative to the Fermi level, the higher in energy the antibonding states and the stronger the bond will be (Figure 3). Stamenkovic et al., for example, investigated the ORR activity of various Pt_3_M (M = Ti, V, Fe, Co, and Ni) alloys with Pt-skin and Pt-skeleton surface structures, employing both experimental and computational methods (Stamenkovic et al., 2007). They constructed a volcano plot using experimentally measured activity and theoretically calculated *d*-band center values. This analysis revealed that Pt_3_Co, Pt_3_Fe, and Pt_3_Ni exhibited significantly enhanced ORR activity compared to pure Pt.

The *d*-band center theory can also be applied to low-dimensional electrocatalysts (Wu et al., 2016; Gao G. et al., 2018; Tao et al., 2019; Ye et al., 2020; Jiao et al., 2022). For instance, Ling et al. established correlations between the d-band center and the adsorption strengths of *OH, *O, and *OOH, along with the potential of elementary reactions for OER on *β*_12_ boron monolayer (*β*_12_-BM)-supported single-atom catalysts (SACs) (Ling et al., 2017). Ni SACs supported on *β*_12_-BM exhibited a moderate energy level of the *d*-band center, resulting in a lower overpotential for OER compared to the other systems under study. Additionally, they proposed an optimal d-band center at −2.82 eV, at which *β*_12_-BM-supported SACs would theoretically have the lowest overpotential for OER. Deng *et al.* designed a series of N-doped graphene-supported transition metal atom-pair catalysts (TM APCs) models for efficient NRR (Deng et al., 2020). Twenty kinds of transition metal atoms were systematically studied and were proven by COHP and orbital interaction analysis; the *d*-band center can be used as a descriptor to describe the NRR performance of the TM APCs. For defective graphene-supported Fe and Al nanoparticles, Lim and co-workers found that the *d*-band center of the Fe and Al nanoparticles shifts closer to the Fermi level, indicating a potential increase in the catalytic reactivity associated with the graphene surface. Zhou *et al.* systematically explored the HER activity of transition metals, transition metal oxides, and carbide substrates covered by nitrogen-doped graphitic sheets and found that the HER activity is correlated to the C *p*_*z*_-band center, which is similar to the *d*-band center theory and determined in turn by the degree of electronic coupling between the graphitic sheet and the metal substrate, enabling the rational design of high-performance hybrid graphitic carbon/transition metal electrocatalysts (Zhou et al., 2018).

#### 3.1.2 Fermi softness

**FIGURE 4:**
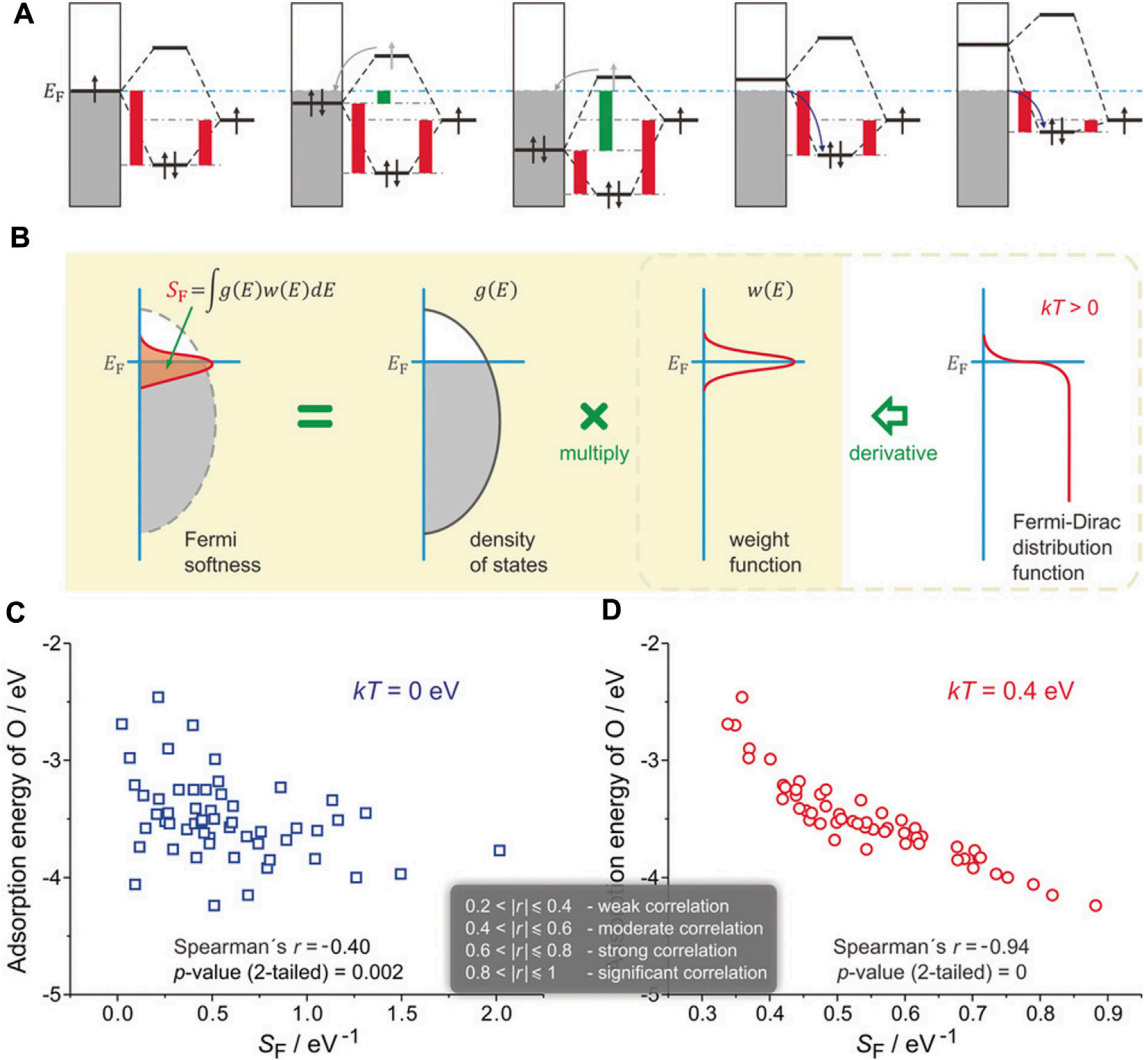
Definition of Fermi softness (*S*_*F*_) and its correlation with surface reactivity. **(A)** Energy analysis of surface bonding. **(B)** S_F_ is defined as a weighted sum of the density of states. **(C)** S_F_ that without and **(D)** with spreading. Reproduced with permission (Huang et al., 2016). Copyright 2016, John Wiley and Sons.

When oxygen atoms are adsorbed on Pt_3_Y (111) surface, even though the surface *d*-band center of Y is higher than that of Pt, the binding strength of Pt-O is stronger than Y-O, defying the *d*-band center theory. However, it can be reasonably explained by the “Fermi softness” (*S*_*F*_), which was developed by Huang and co-workers to describe the electronic structures of a solid surface (Huang et al., 2016). All the bands’ occupied and unoccupied states contribute to the bonding, but those closer to the Fermi level (*E*_*F*_) are more significant (Figure 4A). A reactivity weight function *w*(*E*) with a Fermi level peak is created to determine a weighted summation of the density of states of a solid surface ( $\int g\left(E\right)w\left(E\right)dE$ ) (Figure 4B). The resultant property is the finite-temperature chemical softness, known as *S*_*F*_, which is an accurate descriptor of the surface reactivity. This property is obtained when such a weight function is defined as the derivative of the Fermi-Dirac distribution function at a certain non-zero temperature. For example, the *S*_*F*_ is identical to the density of states at the Fermi level (*g*(*E*_*F*_)) without spreading (i.e., *kT* = 0 eV), which does not closely resemble the surface reactivity (Figure 4C). Then, the *S*_*F*_ displays a substantial association with the surface reactivity when the nominal electronic temperature is changed to 0.4 eV (Figure 4D).

Fermi softness can also be an efficient descriptor to predict the catalytic performance of carbon-based catalysts (Gao R. et al., 2018; Gao Z. et al., 2018; Yang et al., 2018; Xiong et al., 2020; Wang and Zhang, 2022). Gao and co-workers designed a series of catalysts named Fe/GS, which are composed of Fe atom that serves as the active site and different graphene such as single and double vacancy graphene that serve as the substrate (Gao Z. Y. et al., 2018). They further found that the Fermi softness of Fe/GS and the O_2_ adsorption energy exhibit a strong positive linear correlation (*R* ^*2*^ = 0.81) at *kT* = 1.15 eV. The Fermi softness is also used to describe the adsorption activity of Mn-modified graphene catalyst by Wu’s group (Xie et al., 2022). They discovered a substantial negative correlation (*R* ^*2*^ = 0.93) between Fermi softness and adsorption energy at *kT* = 2.8 eV. The Fermi softness is increased with the increase of the adsorption energy.

#### 3.1.3 Energy-based descriptors

Adsorption-free energies (ΔG) of key intermediates are utilized as activity descriptors for recognized reactions, including ΔG_H_ for HER (Han et al., 2023b), ΔG_O_ for ORR (Han et al., 2023a), ΔG_CO_ for CO_2_RR (Chen et al., 2022a), and ΔG_N_ for NRR (Fang et al., 2023). For instance, DFT calculations were conducted on the MoS_2_ edge, yielding a ΔG_H_ of 0.08 eV, which closely approaches the optimal value of 0 eV (Hinnemann et al., 2005). Subsequently, experimental confirmation established that MoS_2_ edges serve as the catalytically active sites for HER (Jaramillo et al., 2007). Through the calculation of free energies of N_2_ adsorption and the formation energy of the key intermediate *NCON, Kou et al. found that V_2_N_6_ sites efficiently catalyze the cleavage of N-N bonds and the coupling of C-N bonds, facilitating effective urea production (Liu J. et al., 2023). Through calculating ORR energy profiles on various active sites within defective graphene, including pyridinic N sites, adjacent carbon atoms, and edge-located pentagon defects, Yao and colleagues determined that pentagon defects serve as the primary active sites for acidic ORR. Subsequent experimental studies have also validated these findings (Jia et al., 2019).

In multi-step catalytic processes, the adsorption and desorption of the intermediates occur during the concerted proton–electron transfer steps, and the adsorption energies of the intermediates follow a linear scaling relationship (Man et al., 2011; J. K. Nørskov et al., 2005; Nørskov et al., 2004). For example, the relationship between the adsorption energies of the intermediates of OER and ORR, such as *O, *OH, and *OOH, can be presented by:

$$∆G_{2}^{\left(i\right)}=A_{1,2}^{\left(i\right)}∙∆G_{1}^{\left(i\right)}+B_{1,2}^{\left(i\right)}$$ (3.1)

Where $∆G_{1}^{\left(i\right)}$ and $∆G_{2}^{\left(i\right)}$ represent the chemisorption energies of oxygen intermediates and $A_{1,2}^{\left(i\right)}$ and $B_{1,2}^{\left(i\right)}$ represent the slope and intercept, respectively, derived from the fitting of adsorption energy data (Abild-Pedersen et al., 2007; Calle-Vallejo et al., 2015a). Fundamentally, the quantity of valence electrons of the atoms attached to the surface has a significant impact on the slope of Eq. (3.1) on closed-packed and low-index surfaces. Since the oxygen atom in OH* only requires one electron to comply with the octet rule, whereas O* requires two, the slope between the adsorption energies of *OH and *O is roughly 1/2. Scaling relationships offer a possible solution to use a few descriptors to fully present the numerous factors influencing a catalytic reaction. Combined with the Sabatier principle, volcano curves can be derived from this linear relationship to reveal the connection between structures and performance. In many cases, $∆G_{OH}$ and $∆G_{O}-∆G_{OH}$ are considered as the descriptors for ORR and OER, respectively (Zheng et al., 2015; Seh et al., 2017; Mao et al., 2018). According to the statistical findings, the free energy difference for monodentate adsorbates can be concluded as $∆G_{OOH}=∆G_{OH}+3.2\;\pm \;\left(0.2\right)$ eV (Man et al., 2011).

#### 3.1.4 Multiple descriptors

The excellent descriptors include *e*_*g*_-filling (Matsumoto et al., 1977a; Matsumoto et al., 1977b), average O-*2p*-state energy ( ${\bar{\varepsilon }}_{2p}$ ), (Lee et al., 2011; Grimaud et al., 2013) surface distortion (Chattot et al., 2018), and generalized coordination numbers ( $\bar{CN}$ ). (Calle-Vallejo et al., 2014; Calle-Vallejo et al., 2015b) Normally, a single descriptor cannot completely predict the performance and synergistic effect of complicated mixed-phase catalysts. Some interface parameters that can demonstrate or influence electron distribution reconfiguration, for instance, adsorption energy (Tran and Ulissi, 2018; Guo C. et al., 2019; Wan et al., 2021), charge transfer (Guan et al., 2019; Mao et al., 2021), surface properties (defects/microstructure/facet) (Back et al., 2019; Parker et al., 2020), and bond length (Wexler et al., 2018; Zhu et al., 2019; Zheng et al., 2020), may serve as the descriptors to predict the best catalysts. Furthermore, a variety of descriptors have been used for describing the reactions with the assistance of machine learning and high-throughput calculations.

**FIGURE 5:**
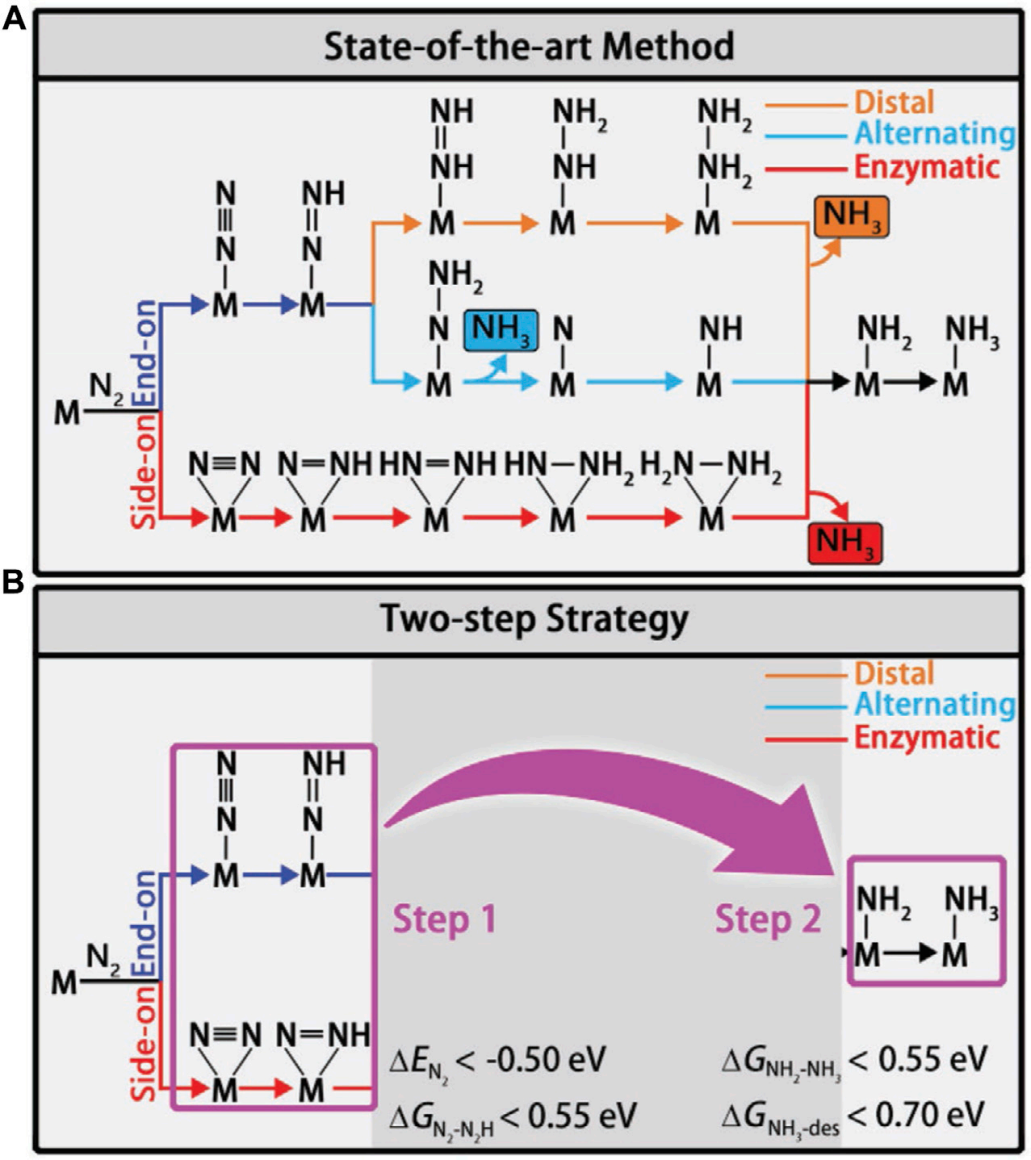
**(A)** Schematic depiction of different mechanisms for N_2_ reduction. **(B)** Flowchart of the two-step screening procedure for NRR catalysts. Reproduced with permission (Ling et al., 2018a). Copyright 2018, John Wiley and Sons.

Different reaction mechanisms can be used to explain the complex NRR, including distal, alternating, and enzymatic (Figure 5A). To identify the viable routes and theoretical onset potentials by the classical approach, all chemical intermediates will be systematically investigated, which will be inefficient for large-scale catalyst screening. Ling *et al.* focused on two “stable to instable” transitions in the NRR process (Eq. 3.2 and 3.3). They set up a two-step method (Figure 5B) to efficiently and accurately screen the nitride-doped-graphene-supported single-atom catalysts (N-G-supported SACs) (Ling et al., 2018a).

$$*N_{2}+H^{+}+e^{-}=*N_{2}H$$ ( 3.2)

$$*{NH}_{2}+H^{+}+e^{-}=*{NH}_{3}$$ (3.3)

In the first step, the systems with low activity are disregarded by using the descriptors $∆E_{N_{2}}$ (adsorption energy of N_2_) and $∆G_{N_{2}-N_{2}H}$ (free energy change of Eq. (3.2)). In the second step, among the systems that meet the requirements of the first step, high-performance catalysts are sought after using the descriptors $∆G_{{NH}_{3}-des}$ (free energy for NH_3_ desorption) and $∆G_{{NH}_{2}-{NH}_{3}}$ (free energy change of Eq. (3.3)). Out of 540 N-G-supported SAC systems, 10 interesting candidates with high NRR catalytic activity were selected using the two-step high-throughput screen approach. For example, W_1_C_3_ demonstrates the best performance with a low onset potential of 0.25 V. Zhou and co-workers also used multiple descriptors to predict the NRR performance of transition metal atoms filled with boron nitride nanotubes (BNNTs) (Zhou et al., 2020). The diameter of BNNTs, work function, and electron transfer from metal to BNNTs, which have negative correlations with the adsorption energy of N_2_, can serve as efficient descriptors to select highly active catalysts.

### 3.2 Application of theoretical calculations in revealing the reaction mechanism

With the development of modern characterization techniques, an increasing number of *in situ* characterization tools are used to observe the catalytic reaction processes, aiming to accurately uncover the catalytic mechanism. However, it is still difficult to identify the actual active site and reaction intermediates of complex catalytic reactions by pure experimental study. In terms of CO_2_RR, through different reaction routes, the products of CO_2_RR can be carbon monoxide, formic acid, ethylene, or ethanol. Depending on whether the adsorption sites change or not during the catalytic processes, the actual reaction paths and intermediates and the competition between the carbon-carbon coupling reaction and HER are all unknown and difficult to elucidate experimentally (Zhang et al., 2021). For instance, Dual-atom catalysts (DACs) have been considered promising candidates for C_2_ product generation due to their ability to offer two metal sites that enhance *CO coverage on the surface. However, experimental results indicate that most DACs exhibit high Faradic efficiency for CO, while the formation of multi-carbon products is seldom observed (Gong et al., 2022; Hao et al., 2022; Zhao et al., 2022). Li and colleagues conducted DFT calculations and discovered a deviation from the conventional hypothesis, suggesting that C−C coupling typically does not occur at the metal-top sites (Yang et al., 2023). Surface Pourbaix analyses indicate that CO preferably occupies the bridge sites between two metals, potentially impeding subsequent C−C coupling reactions. Furthermore, based on energy variations, it is not feasible for C−C coupling to occur on the surface of a DAC, both in terms of thermodynamics and kinetics. In experimental settings, oxide-derived copper (OD-Cu) has demonstrated exceptional performance, displaying remarkable selectivity toward C_2+_ products even at low potentials (Cheng et al., 2021; Chen et al., 2022b). Nevertheless, the understanding of the atomic structures of active sites in OD-Cu remains limited due to its inherent complexity. Cheng et al. conducted multiscale computations based on first principles to simulate the synthesis, characterization, and performance assessment of Cu nanoparticles deposited on carbon nanotubes (CNTs) (Cheng et al., 2017). Their investigation identified two active sites that show an undercoordinated surface square structure adjacent to a subsurface stacking fault and exhibit lower formation energies for *COCOH compared to the Cu (100) surface sites.

Meanwhile, theoretical calculation is a key method to compensate for experimental shortcomings because it can investigate the electronic-scale change during the catalytic reaction. In the past decades, the use of theoretical simulations in defective electrocatalysis has greatly aided the catalytic mechanism analysis of novel catalysts (Hu et al., 2021; Khan et al., 2021; Lan et al., 2021; Liu A et al., 2021).

**FIGURE 6:**
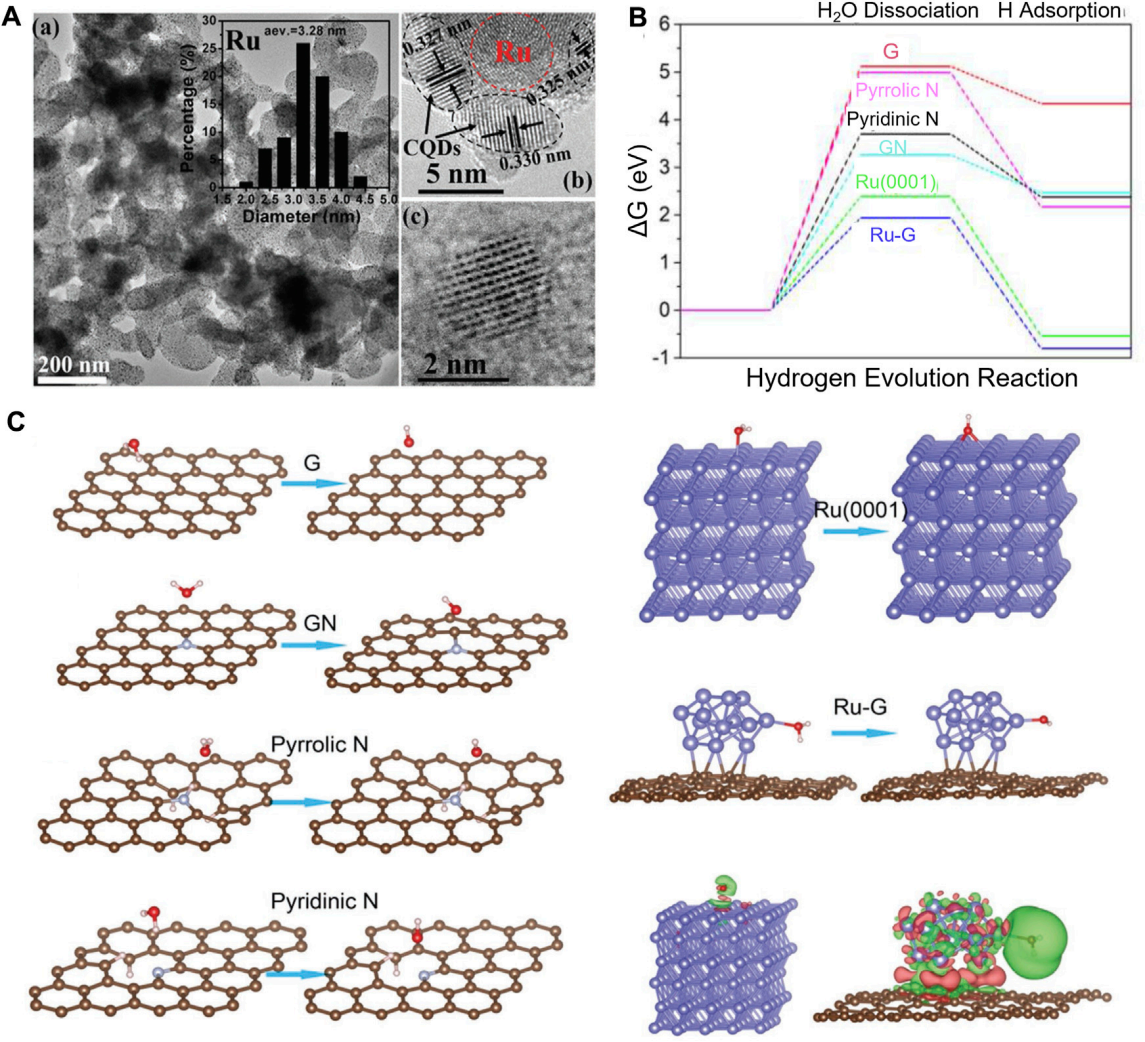
**(A)** TEM and HRTEM images of Ru@CQDs. **(B)** Calculated HER relative energy diagram. **(C)** Adsorption and dissociation of the H_2_O molecule on different surfaces. Reproduced with permission (Li M. et al., 2018). Copyright 2018, John Wiley and Sons.

With the assistance of DFT calculations, Zhang *et al.* systematically investigated the origins of the high-performance atomic Co-Pt embedded into nitrogen-doped graphene (A-CoPt-NC) (Zhang B-W et al., 2018). A stable adsorption state of the intermediate in the HER is proved by the electron distribution calculations. The disappearance of charge depletion on the surface of the outer layer can improve the adsorption of protons to the catalyst. Li and co-workers synthesized a new HER electrocatalyst (Li M. et al., 2018). Carbon quantum dots were used to support ruthenium nanoparticles (Ru@CQDs). It shows superior catalytic activity and durability in alkaline conditions. DFT calculations provide compelling evidence of the excellent HER catalytic activity of Ru supported in the N-doped CQDs layer. The calculation results show the synergistic effect of doped N atoms and Ru clusters, and both are beneficial in reducing the dissociation energy of H_2_O. The electron transfer from Ru and H_2_O to C atoms, which is proved by the difference charge density analysis, can polarize Ru clusters, thus contributing to the dissociation of H_2_O. Meanwhile, the dissociated H atoms are held together by Ru and H interactions to form the H_2_ molecule (Figure 6).

As can be seen from the above discussions, theoretical calculation is an indispensable method to investigate the relationship between structures and performance. A reaction model will be built to thoroughly study the basic reaction path of the reaction, identify the crucial steps that determine the reaction rate, and then optimize the chemical reaction conditions. It is obvious that theoretical calculations are beneficial in identifying the basic process and reaction paths of electrocatalysis at the atomic level. Furthermore, the design of catalysts and the controllable synthesis of the desired structures can be aided by the DFT simulations, which can be utilized as a prediction tool. Theoretical modelings can also be used in catalyst structure optimization and validation of the design outcomes, as well as to support experimental study.

## 4 Recent advances in computational studies on low-dimensional carbon-based electrocatalysts

### 4.1 Hydrogen evolution reaction (HER)

Hydrogen is one of the most promising clean energy sources to replace fossil fuels, and it also has the highest energy density among existing fuels. Water splitting is a sustainable way of producing hydrogen compared to industrial reduction of natural gas (CH_4_). Hydrogen evolution reaction (HER) is the cathodic half-cell reaction of water electrolysis (Karunadasa et al., 2010). The large overpotential restricts its practical application and high-performance electrocatalysts are needed to boost the HER. Noble metal-based catalysts such as Pt/C and RuO_2_ are commonly considered to be the most efficient HER electrocatalysts; however, scarcity and low durability limit their mass industrial production (Andreadis and Tsiakaras, 2006; Hao et al., 2016). To date, many high-performance low-dimensional carbon-based non-noble metals and even metal-free electrocatalysts have been designed and fabricated to be the alternatives of noble metal materials. In this part, the fundamental concepts and recent developments in HER electrocatalysts will be summarized.

HER is a two proton-electron transfer step (PETS) process. The first step is the Volmer reaction, i.e., the adsorption of hydrogen, which is presented in Eq. (4.1a) (acidic condition) and 4.1b (alkaline condition).

$$H^{+}+e^{-}+*\;\to \;H^{*}$$ (4.1a)

$$H_{2}O+e^{-}+*\;\to \;H^{*}+OH^{-}$$ (4.1b)

The second step can be the Heyrovsky reaction or the Tafel reaction. Equation (4.2a) (acidic condition) and 4.2b (alkaline condition) show the Heyrovsky reaction and Eq. (4.3) shows the Tafel reaction.

$$H^{*}+H^{+}+e^{-}\;\to \;H_{2}+*$$ (4.2a)

$$H^{*}+H_{2}O+e^{-}\;\to \;H_{2}+{OH}^{-}+*$$ (4.2b)

$$2H^{*}\;\to \;H_{2}+*$$ (4.3)

In which, * represents the active site on the catalyst surface.

**FIGURE 7:**
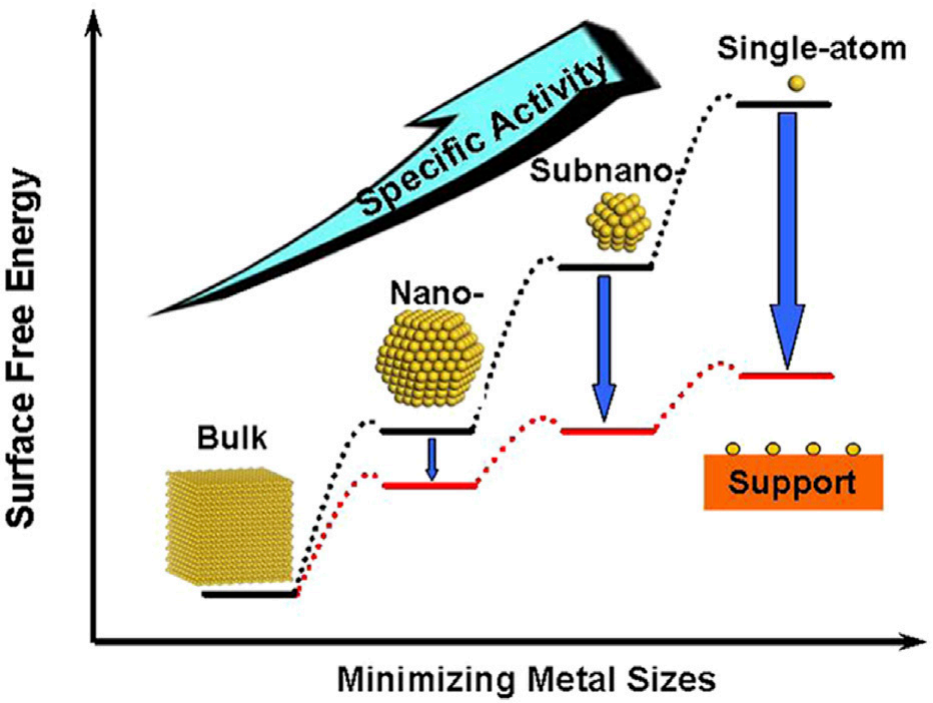
Schematic illustration of the changes of surface-free energy and specific activity per metal atom with metal particle size and the support effects on stabilizing single atoms. Reproduced with permission (Yang et al., 2013). Copyright 2013, American Chemical Society.

For the design of low-cost and efficient electrocatalysts, it is crucial to reduce the amount of precious metals being used, which can be achieved in two ways. Firstly, the specific activity per metal atom usually increases with the downsize of metal particles (Figure 7). (Yang et al., 2013) Therefore, single-atom catalysts (SAC) that contain isolated metal atoms singly dispersed on supports have attracted extensive research attention. Alternatively, non-noble metal atoms or functional groups can also be anchored on the substrate to tune the electronic structures of noble-metal active sites (Sun et al., 2013; Gao G. et al., 2018; Li W. et al., 2018; Ren et al., 2020; Yang et al., 2022). Ye and co-workers designed a novel Pt SAC using aniline-stacked graphene as the support (Pt SASs/AG). It shows excellent HER performance with *η* = 12 mV at 10 mA cm^−2^ and a mass current density of 22,400 ${Ag}_{Pt}^{-1}$ at *η* = 50 mV, which is 46 times higher than that of the commercial 20 wt% Pt/C (Ye et al., 2019). Moreover, the Pt SASs/AG catalyst presents outstanding stability. With the assistance of DFT calculations, they found that the interaction between the atomical Pt and the nitrogen of aniline makes the *d*-band center of Pt downshift to −2.465 eV, which is close to that of Pt (111) (−2.687 eV). Additionally, the density of states (DOS) near the Fermi level of Pt in Pt SASs/AG catalyst is as large as that of Pt in Pt (111), eventually promoting the HER activity.

**FIGURE 8:**
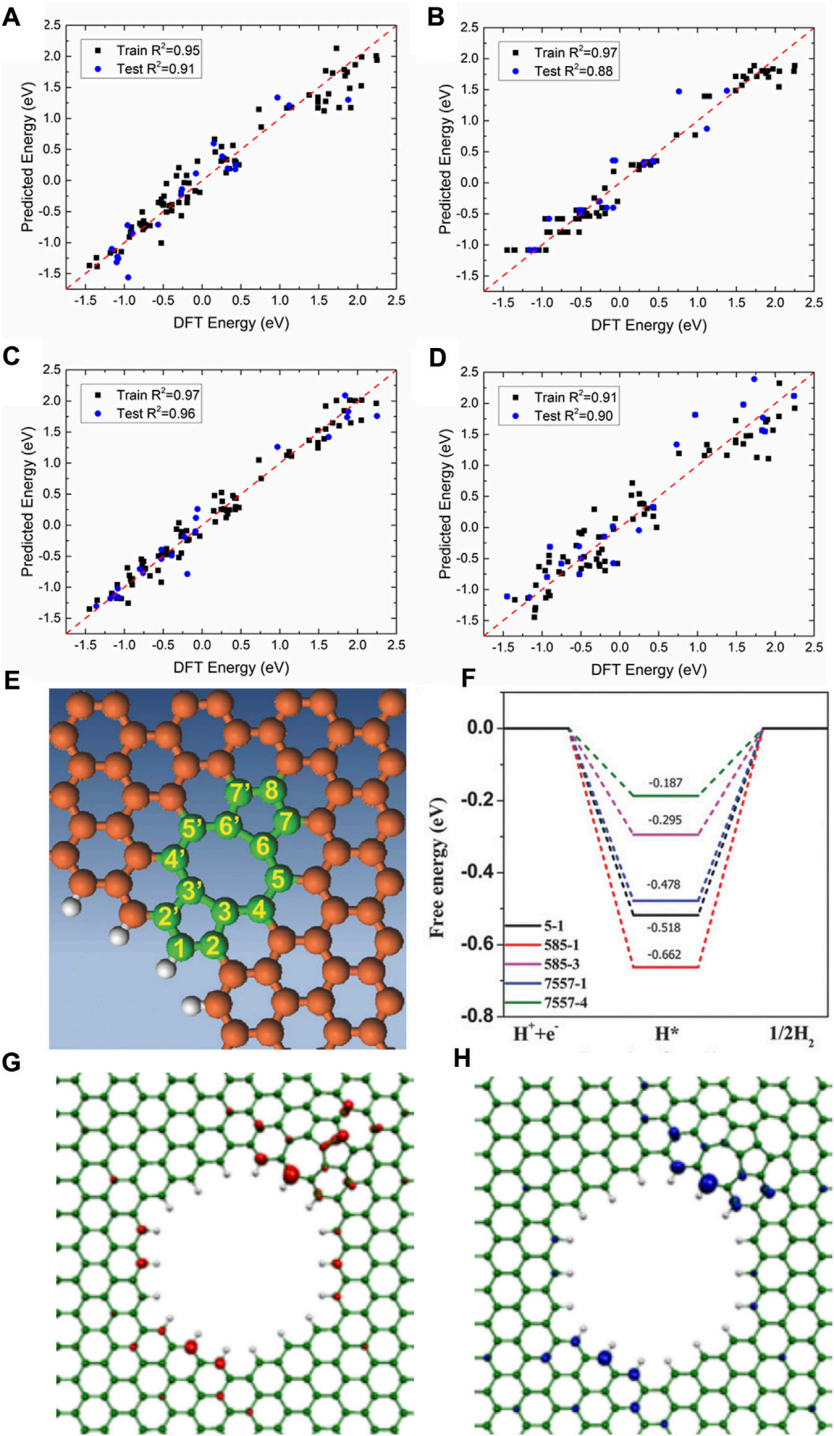
Comparison of DFT-calculated *versus* machine learning-predicted $∆G_{H}$ using **(A)** kernel ridge regression, **(B)** random forest regression, **(C)** neural network regression, and **(D)** SISSO regression. Reproduced with permission (Fung et al., 2020). Copyright 2020, American Chemical Society. **(E)** Mechanism study model of 7557defect. **(F)** Schematic energy profiles for the HER pathway. **(G)** HOMO and **(H)** LUMO orbitals for 7557 defect. Reproduced with permission (Jia et al., 2016). Copyright 2016, John Wiley and Sons.

Secondly, non-noble metals such as transition metals are used as efficient HER catalysts, and even metal-free catalysts with excellent performance were designed and synthesized experimentally (Fan et al., 2016; He et al., 2017; Zhang L et al., 2018; He et al., 2019; Ahsan et al., 2021; Yasin et al., 2022; Han et al., 2023b; Yin and Du, 2023). For example, using the first principle DFT calculations, 3D, 4D, and 5D transition metal SACs in N-doped 2D graphene and nanographene of various sizes are screened for HER by Fung *et al.* (Fung et al., 2020) For most SACs, a *d*-band center downshift will occur when moving from graphene to nanographene, indicating that the hydrogen adsorption on metal SACs can be tuned by adjusting the size and dimension of the substrate. V, Rh, and Ir embedded in N-doped nanographene show much better HER activity than those on the extended 2D graphene. The machine learning models (kernel ridge regression, decision trees (random forest), and neural networks) and the SISSO (Sure Independence Screening and Sparsifying Operator) method are employed to accurately predict the $∆G_{H}$ using various proposed descriptors; the results are shown in Figures 8A–D. Topological defect-based and complex defect-based carbon materials are also promising electrocatalysts. Yao and co-workers synthesized a series of defective carbons via a facile nitrogen removal procedure from N-doped graphene, and the edge-defect model called 7557-4 shows outstanding HER performance with the lowest calculated free energy of 0.187 eV (Figures 8E,F). (Jia et al., 2016) DFT calculations were also performed to better understand the underlying catalytic mechanisms. The analysis of the frontier molecular orbitals shows that the catalytic activity of edge atoms is laid on their most contribution to the highest occupied molecular orbital (HOMO) and lowest unoccupied molecular orbital (LUMO), which are highly correlated with the catalytic reactions (Figures 8G,H).

### 4.2 Oxygen evolution reaction (OER) and oxygen reduction reaction (ORR)

Oxygen evolution reaction (OER, $2H_{2}O\;\left(l\right)\;\to \;O_{2}\;\left(g\right)+4e^{-}+4H^{+}$ ) is the anode half-cell reaction of water splitting containing four electron transitions, as shown in Eq. 4.4. Oxygen reduction reaction (ORR, $O_{2}\;\left(g\right)+4e^{-}+4H^{+}\;\to \;2H_{2}O\;\left(l\right)$ , Eq. 4.5) is the revise reaction of the OER, which is the cathodic reaction of fuel cells and zinc-air batteries (Gasteiger et al., 2005).

$$H_{2}O\;\left(l\right)+*\;\to \;OH^{*}+e^{-}+H^{+}$$ (4.4a)

$$OH^{*}\;\to \;O^{*}+e^{-}+H^{+}$$ (4.4b)

$$O^{*}+H_{2}O\;\left(l\right)\;\to \;OOH^{*}+e^{-}+H^{+}$$ (4.4c)

$$OOH^{*}\;\to \;O_{2}+e^{-}+H^{+}$$ (4.4d)

$$O_{2}+e^{-}+H^{+}\;\to \;OOH^{*}$$ (4.5a)

$$OOH^{*}+e^{-}+H^{+}\;\to \;O^{*}+H_{2}O\;\left(l\right)$$ (4.5b)

$$O^{*}+e^{-}+H^{+}\;\to \;OH^{*}$$ (4.5c)

$$OH^{*}+e^{-}+H^{+}\;\to \;H_{2}O\;\left(l\right)+*$$ (4.5d)

**FIGURE 9:**
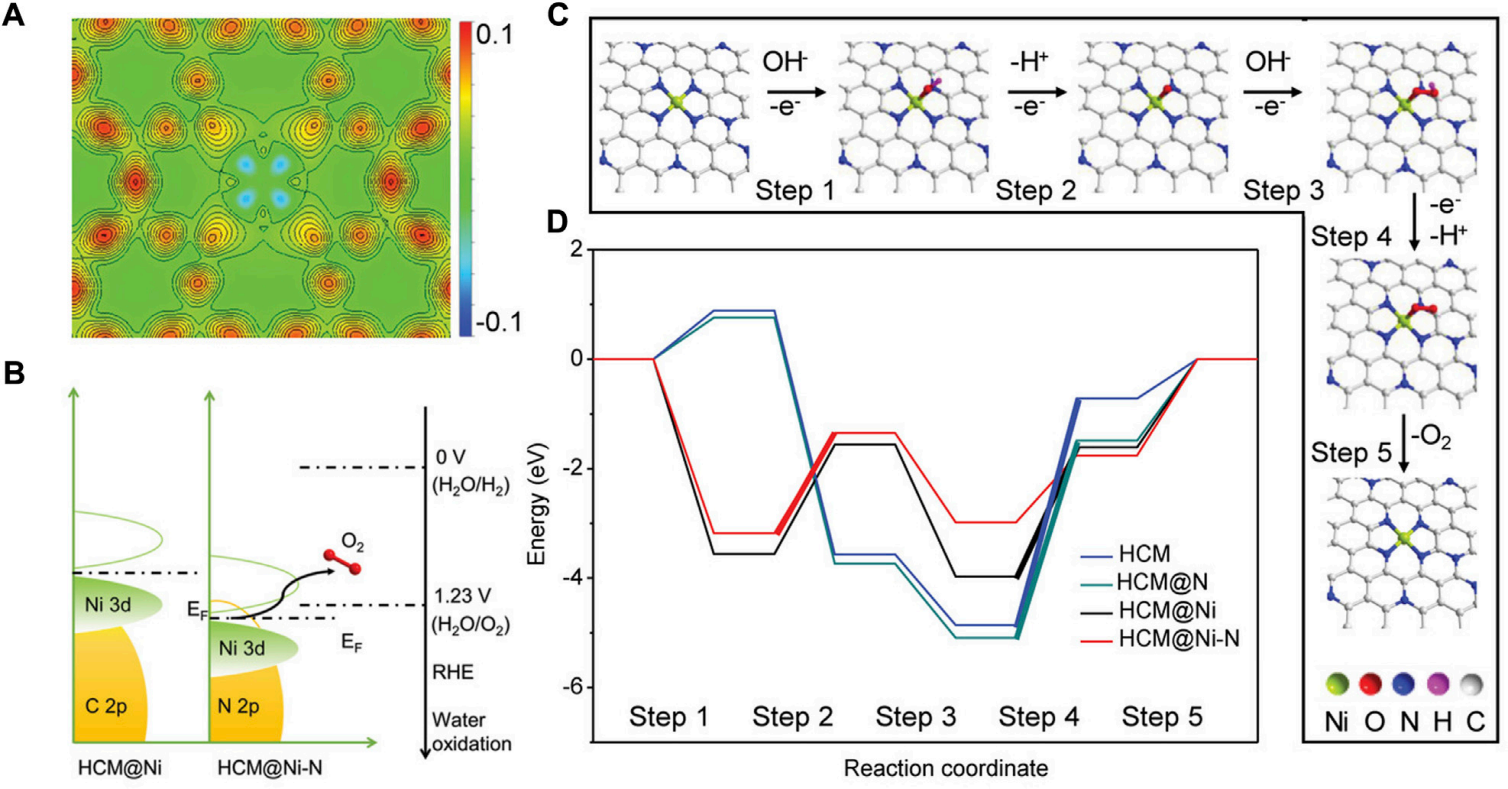
**(A)** Calculated distribution of charge density for HCM@Ni-N. **(B)** Schematic band diagrams of HCM@Ni and HCM@Ni-N. **(C)** Proposed mechanism of O_2_ evolution over HCM@Ni-N. **(D)** Free energy diagram at 0 V for OER over HCM, HCM@N, HCM@Ni, and HCM@Ni-N. The bold lines indicate the rate-determining step. Reproduced with permission (Zhang et al., 2019). Copyright 2019, John Wiley and Sons.

Both the OER and ORR are restricted by the sluggish kinetic and high reaction energy barriers, thus electrocatalysts are needed to accelerate their reaction rates. However, the high cost and low abundance of current commercial noble metal-based catalysts, i.e., Pt/C for ORR, RuO_2_ and IrO_2_ for OER severely hinder their widespread applications. Similarly, the strategies of lowering the cost and improving the performance of the HER catalysts are also applicable to the OER and ORR electrocatalysts, and great achievements have been accomplished (Zhu et al., 2018; He et al., 2020a; Wang et al., 2020a; Yan et al., 2020; Yang et al., 2020; Yan et al., 2021; Xu et al., 2023). Zhang *et al.* anchored atomical distributed Ni atoms onto an N-doped hollow carbon matrix (HCM@Ni-N) (Zhang et al., 2019). In alkaline conditions, the HCM@Ni-N only requires an OER overpotential of 304 mV to reach the current density of 10 mA cm^-2^, which is much lower than that of the RuO_2_ (393 mV), suggesting excellent OER activity of the prepared catalyst. Through the calculated distributions of charge density, they found that the electronic distribution of N-doped HCM was changed obviously after the Ni decoration (Figure 9A). In addition, the *3d* orbital of Ni in HCM@Ni-N shows a leftward shift, and the *d*-band center of the Ni was downshifted from −0.94 eV to −2.04 eV as a result of the Ni-N interaction (Figure 9B). According to the *d*-band center theory, this change could facilitate the desorption of adsorbates and reduce the energy barrier. The free energy pathways of the OER can also be calculated by DFT simulations and they are in good agreement with the experimental results (Figures 9C,D). By encapsulating Co and *β*-Mo_2_C into N-doped carbon nanotubes (Co/*β*-Mo_2_C@N-CNT), Ouyang and co-workers successfully fabricated a bifunctional electrocatalyst for the HER and OER in an alkaline electrolyte (Ouyang et al., 2019). Based on the heterointerface between Co and *β*-Mo_2_C, the OER activity of *β*-Mo_2_C is enhanced significantly. With the assistance of DFT calculations, they proved that the joint effect of N-CNTs, Co, and *β*-Mo_2_C resulted in the low energy barriers of the intermediates, thus greatly improving the HER and OER kinetics.

**FIGURE 10:**
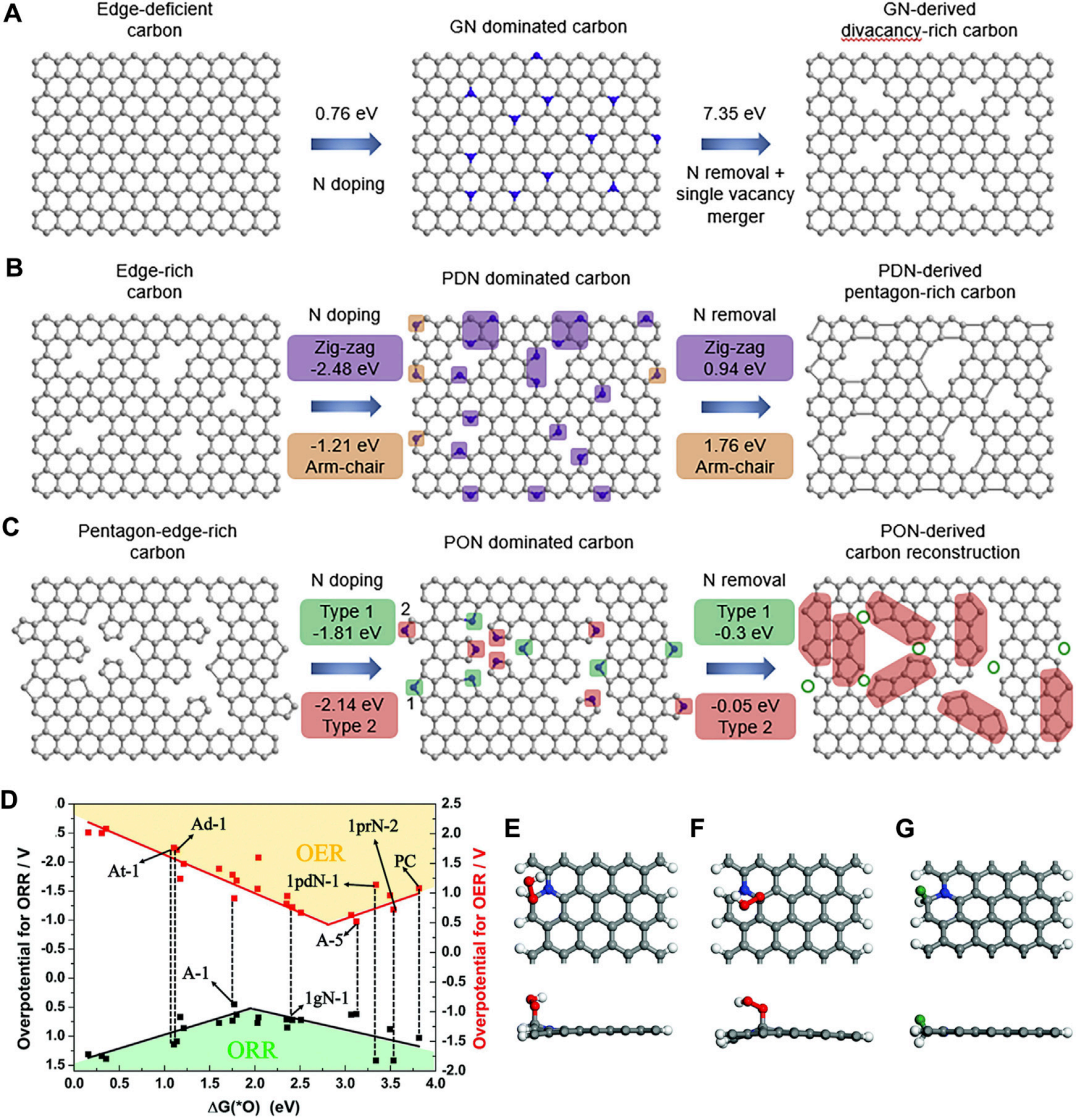
Computational simulation of specific N-doping and removing process in different carbon models: **(A)** Schematic and formation energy calculation of transformation from edge-deficient carbon to GN-dominated carbon and then to divacancy-rich carbon. **(B)** Schematic and formation energy calculations of transformation from edge-rich carbon to PDN-dominated carbon and then to pentagon-rich carbon. **(C)** Schematic and formation energy calculations of transformation from pentagon-edge-rich carbon to PON-dominated carbon and then to special carbon reconstruction. Reproduced with permission (Wang et al., 2020b). Copyright 2020, Elsevier. **(D)** The volcano plot for the ORR and OER by plotting the overpotential as a function of $∆G\left(*O\right)$ at various possible active sites. The top and side views of the active site **(E)** A-1 for the ORR, **(F)** A-3 for the OER with OOH adsorbed, and **(G)** A-1 for the HER; the green ball represents the adsorbed H (*θ* = 2.27%). Reproduced with permission (Jiang et al., 2019). Copyright 2008, Royal Society of Chemistry.

Under the guidance of DFT calculations, Wang *et al.* successfully synthesized a series of topological carbon defects through a facile N-removing strategy, among which adjacent pentagons (A-C5) show the best catalytic performance for the ORR and the edge divacancy defects (C585-2) are favorable for the HER (Wang et al., 2020b). DFT calculations were applied to investigate the relationship between the original carbon structure, the type of N configuration obtained, and the corresponding defect structures, for example, perfect carbon network *versus* graphitic-N *versus* C585 (Figure 10A), edge-rich hexagonal structure *versus* pyridinic-N *versus* S-C5 (Figure 10B), and edge-rich pentagon *versus* pyrrolic-N *versus* A-C5 (Figure 10C). By a spontaneous gas-foaming method, Jiang and co-workers fabricated a range of promising trifunctional electrocatalysts named defect-rich N-doped ultrathin carbon nanosheets for HER, OER, and ORR (Jiang et al., 2019). In rechargeable Zn-air batteries, NCN-1000-5 exhibits a high energy density (806 Wh/kg), a low charge/recharge voltage gap (0.77 V), and an extremely long cycle life (over 300 h). DFT calculations were used to identify the intrinsic active sites for the electrochemical reactions. The catalytic performance of various active sites for the ORR and OER was investigated, and the results are shown in the volcano plot (Figure 10D). The armchair edge carbon atoms, which are adjacent to the graphitic-N, possess the lowest overpotential, thus they should be the optimal catalytic active center for the specific electrocatalysis (Figures 10E–G).

### 4.3 Carbon dioxide reduction reaction (CO_2_RR)

Carbon dioxide reduction reaction (CO_2_RR) could convert CO_2_ to many value-added chemicals, such as CO, CH_4_, CH_3_OH, and C_2_H_5_OH (Eq. 4.6) (Whipple and Kenis, 2010; Chen J. et al., 2021; Chen S. et al., 2021), which can be directly used as fuels to replace fossil fuels like gasoline and natural gas. Therefore, the CO_2_RR could significantly relieve the greenhouse gas effect. However, the hydrogen atoms necessary for the reduction of CO_2_ molecules are transported from the aqueous solution, so the HER must be considered as the competitive reaction. The high cost of the currently used noble metal-based catalysts and the low selectivity of CO_2_RR are still the biggest obstacles to its industrialization. Therefore, developing highly efficient and selective non-noble metal CO_2_RR catalysts based on earth-abundant elements has attracted the most attention in this promising research field (Back et al., 2017; Mao et al., 2020a; He et al., 2020b; Er et al., 2021; Zha et al., 2021; Powar et al., 2022).

$$CO_{2}+e^{-}\;\to \;CO_{2}^{-}\;E^{Ɵ}=-1.90\;V$$ (4.6a)

$$CO_{2}+2H^{+}+2e^{-}\;\to \;CO+H_{2}O\;E^{Ɵ}=-0.52\;V$$ (4.6b)

$$CO_{2}+2H^{+}+2e^{-}\;\to \;HCOOH\;E^{Ɵ}=-0.61\;V$$ (4.6c)

$$CO_{2}+4H^{+}+4e^{-}\;\to \;HCOH+H_{2}O\;E^{Ɵ}=-0.51\;V$$ (4.6d)

$$CO_{2}+6H^{+}+6e^{-}\;\to \;CH_{3}OH+H_{2}O\;E^{Ɵ}=-0.38\;V$$ (4.6e)

$$CO_{2}+8H^{+}+8e^{-}\;\to \;CH_{4}+2H_{2}O\;E^{Ɵ}=-0.24\;V$$ (4.6f)

$$CO_{2}+12H^{+}+12e^{-}\;\to \;C_{2}H_{4}+4H_{2}O\;E^{Ɵ}=-0.34\;V$$ (4.6g)

**FIGURE 11:**
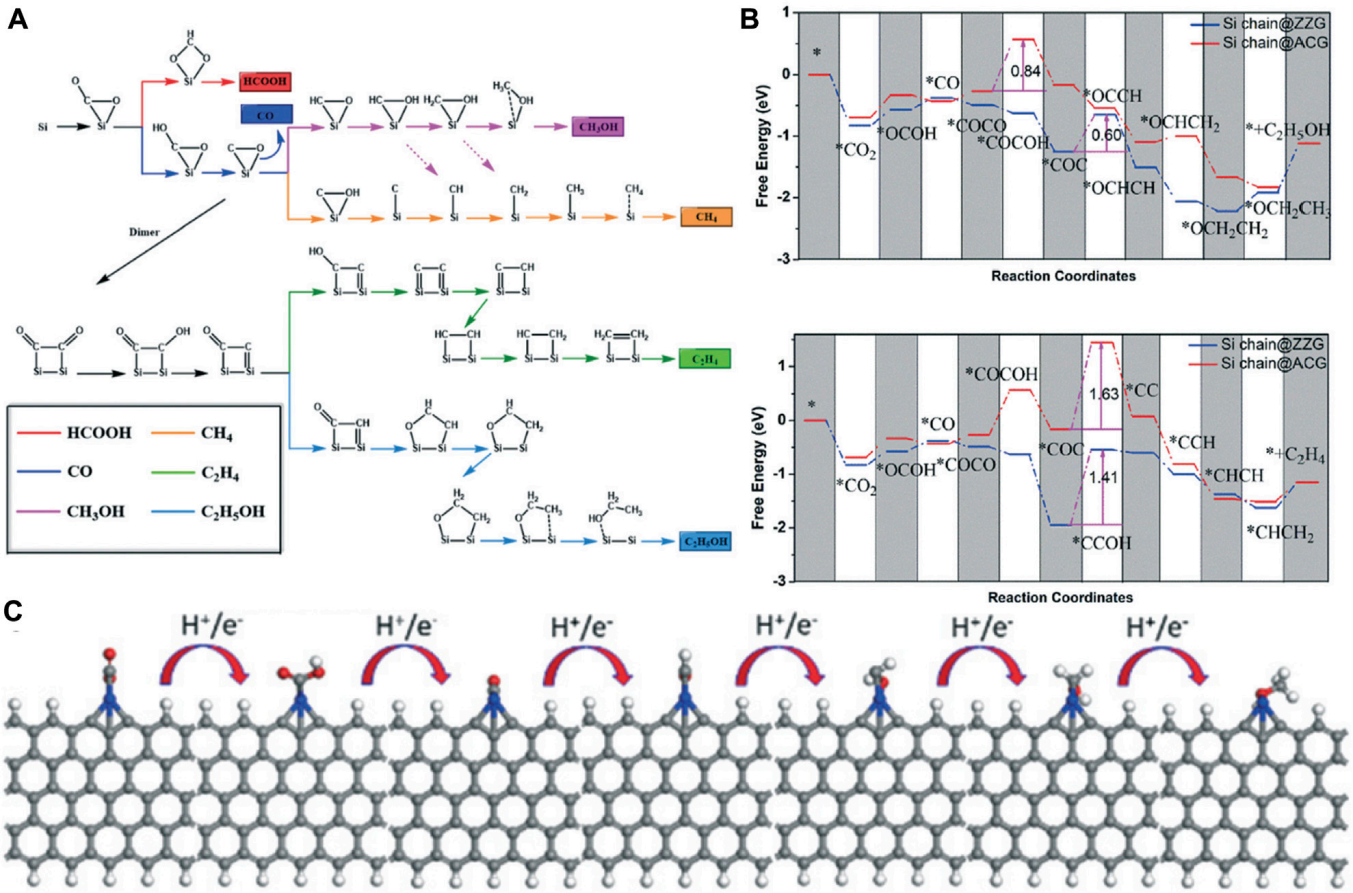
**(A)** Possible reaction pathways for the electrocatalytic reduction of CO_2_ to six different products. **(B)** Gibbs free energy diagrams of CO_2_ reduction reactions to CH_3_OH and C_2_H_4_ on Si chain@ZZG and Si chain@ACG. **(C)** The optimized structures for CO_2_ reduction to CH_3_OH on the zigzag edge. Reproduced with permission (Mao et al., 2019b). Copyright 2011, Royal Society of Chemistry.

The initial stage of CO_2_ reduction is CO_2_ adsorption, which is a crucial step of the CO_2_RR. Zhu *et al.* systematically investigated the adsorption of CO_2_ on the g-C_3_N_4_ surface by DFT calculations (Zhu et al., 2017). Through analyzing the electronic properties such as the band gap, density of states, work function, HOMO, and LUMO, they found that the two-coordinated N atoms, which contributed to both valance band and conduction band edge, have the most negative adsorption energy (−0.4181) for CO_2_ molecule. CO_2_ can be effectively captured and activated on Si due to the “acceptance and back-donation” of electrons between the Si dopant and CO_2_ molecule. Accordingly, Mao and co-workers designed an experimentally synthesizable electrocatalyst called silicon-doped graphene edges (Si chain@G) (Mao et al., 2019b). The catalytic performance of the Si@ZZG (Si atoms doped into the zigzag edge of graphene) and Si@ACG (Si atoms doped into the armchair edge of graphene) was exhaustively studied through DFT calculations (Figure 11A). CO_2_ can be well captured and efficiently activated on Si@G. The binding energy at the zigzag and armchair edges is −0.65 eV and −0.83 eV, respectively. Remarkably, CO_2_ can be converted to CH_3_OH effectively when the Si@ACG is served as the active site. Moreover, Si chain@G with multiple Si active sites which are beneficial to product multiple-carbon productions was also investigated. They found that the Si chain@ZZG has a high selectivity to transform CO_2_ to C_2_H_5_OH with an extreme low limiting potential of −0.6 V under the optimal theoretical reaction pathway (Figures 11B,C).

**FIGURE 12:**
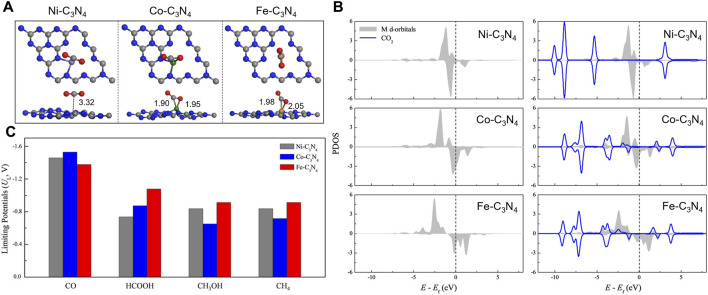
**(A)** The most stable CO_2_ adsorption configurations. **(B)** PDOS of the metal *d* orbital before and after adsorption (including the adsorbent CO_2_ total DOS). **(C)** Summary of the limiting potentials for the productions of CO, HCOOH, CH_3_OH, and CH_4_. Reproduced with permission (Guo X et al., 2019). Copyright 2019, John Wiley and Sons.

Transition metal atoms can also be excellent CO_2_RR active centers. Guo *et al.* established calculation models with Fe, Co, and Ni single atoms embedded onto graphitic carbon nitride (Fe/Co/Ni-C_3_N_4_) and systematically investigated the structures of the electrocatalysts, CO_2_ adsorption configurations, and the reduction mechanisms (Guo X. et al., 2019). g-C_3_N_4_ with six-fold cavities was selected as the substrate, and the introduced Ni, Co, and Fe atoms are located from the corner to the center of the cavity. The PDOS of the metal *d* orbital and adsorbed CO_2_ indicated that CO_2_ could be chemically adsorbed on Co-C_3_N_4_ and Fe-C_3_N_4_ but physically adsorbed on Ni-C_3_N_4_ (Figures 12A,B). Guo *et al.* also probed the reaction pathway and mechanism of different C1 products and thoroughly calculated the limiting potentials for the production of CO, HCOOH, CH_3_OH, and CH_4_. They found that Co-C_3_N_4_ has superior CO_2_RR activity and selectivity for CH_3_OH (Figure 12C). Currently, copper (Cu) is found to be one of the best catalysts for achieving the high activity reduction of CO_2_ to hydrocarbons since *CO_2_ and *COOH can be effectively collected from Cu-based catalysts (Raciti and Wang, 2018). Due to the hydration of non-adsorbing CO_2_ molecules, Pb, Hg, Cd, and Bi also demonstrate good catalytic performance for producing formate (Mao et al., 2020b). Other transition metals such as Ni, Fe, Pd, and Ti exhibit low CO_2_RR selectivity because the HER is much more favorable due to the strong H-bonding (Back et al., 2017).

### 4.4 Nitrogen reduction reaction (NRR)

**FIGURE 13:**
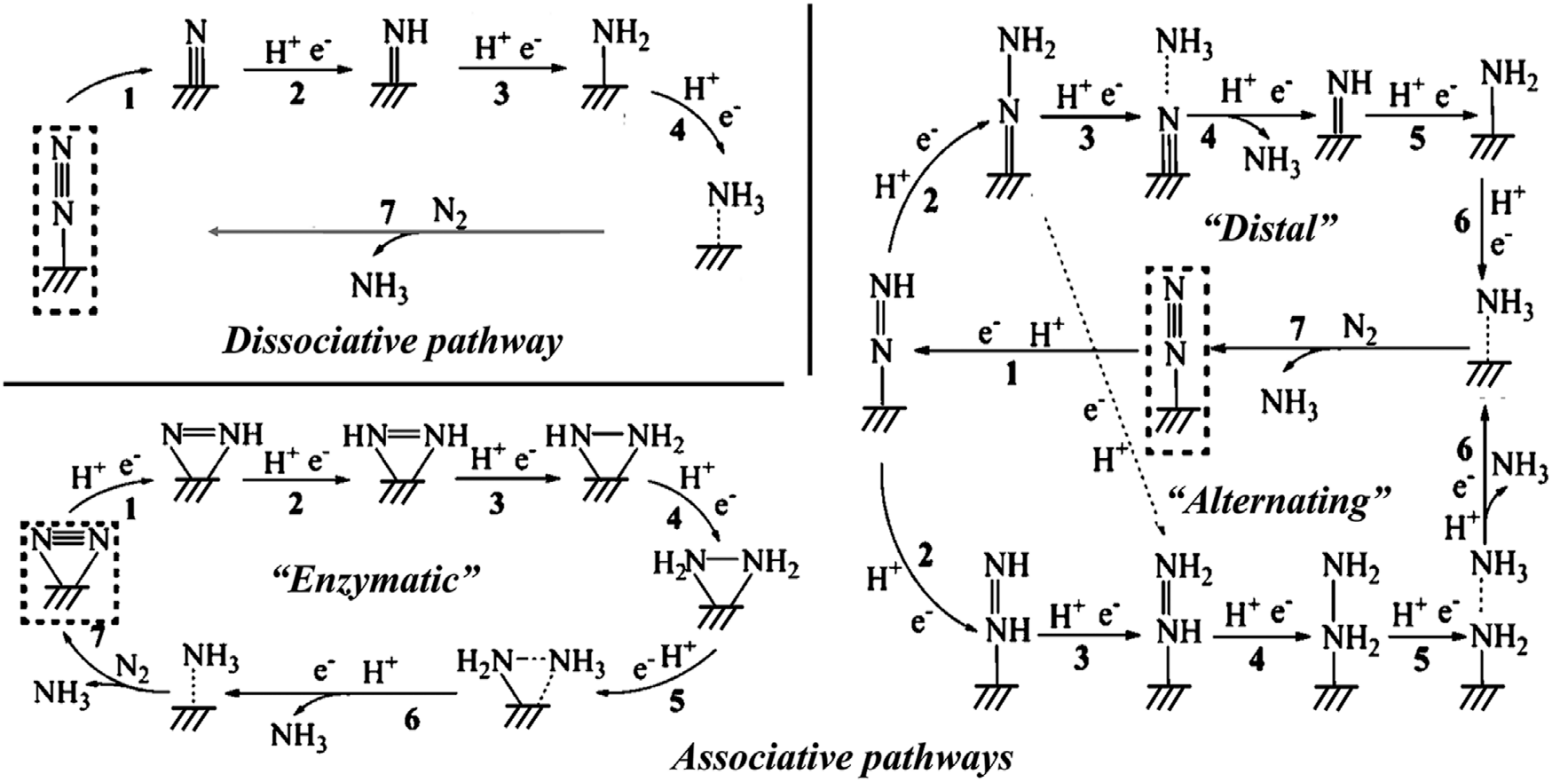
Schematic depiction of the dissociative pathways and the associative pathways (including distal, alternating, and enzymatic pathways) for catalytic conversion of N_2_ to NH_3_. Reproduced with permission (Li et al., 2016). Copyright 2016, American Chemical Society.

Nitrogen is the most abundant element on the earth, and it is essential for all organisms including animals and plants. Ammonia (NH_3_) is one of the most important industrial compounds due to its wide use in different fields. However, the ultra-stable N≡N triple bond greatly impedes the nitrogen fixation reaction. The industrial Haber-Bosch process uses N_2_ and H_2_ as the raw materials, consuming excessive energy because of the high reaction temperature (350°C–550°C) and pressure (150–350 atm) (van der Ham et al., 2014; Chen Jingguang et al., 2018). Therefore, the conversion of N_2_ to NH_3_ under ambient conditions is a promising research area. Recently, electrocatalytic nitrogen reduction reaction (NRR) has attracted increasing research attention because of its obvious advantages such as low energy consumption, reduced reaction temperature, and enhanced productivity (Li et al., 2016; Cui et al., 2018; He et al., 2020c; Liu Y et al., 2021; Ruiyi et al., 2021; Samal et al., 2021; Wang and Mao, 2021; Zhou et al., 2022; Fang et al., 2023; Liu H. et al., 2023). Figure 13 shows the possible reaction pathways of the catalytic conversion of N_2_ to NH_3_.

**FIGURE 14:**
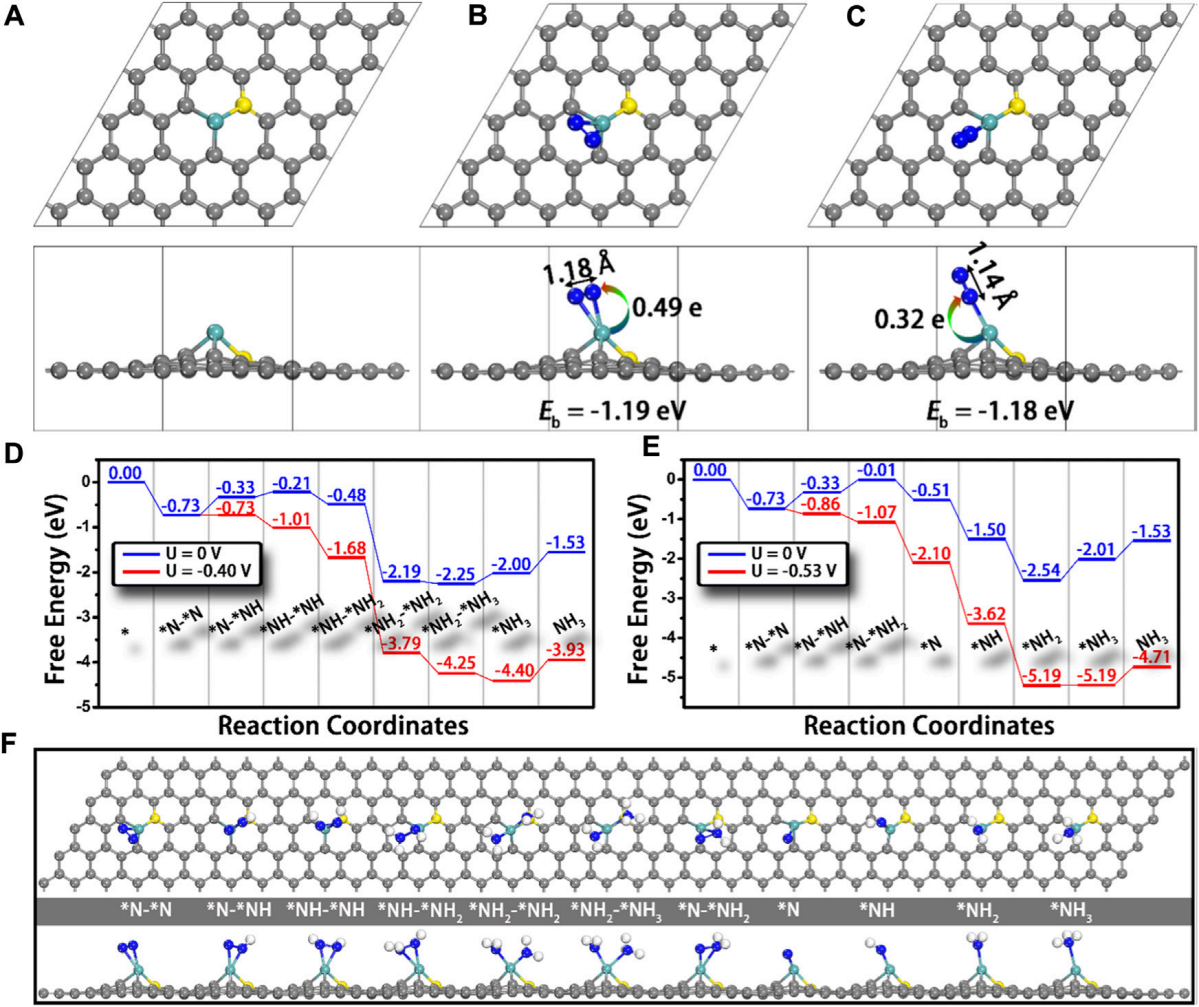
Top and side views of the structures of **(A)** Mo_1_-N_1_C_2_, and Mo_1_-N_1_C_2_ with N_2_ adsorption through **(B)** side-on and **(C)** end-on patterns. N−N bond lengths and charge transfer from Mo_1_-N_1_C_2_ to N_2_ are also presented. Free-energy diagrams for N_2_ reduction through **(D)** enzymatic and **(E)** consecutive mechanisms at different applied potentials as well as **(F)** the corresponding structures of the reaction intermediates. Gray, cyan, yellow, and blue balls represent the C, Mo, doped N, and adsorbed N atoms, respectively. Reproduced with permission (Ling et al., 2018b). Copyright 2018, American Chemical Society.

The first and most important step of the NRR is the adsorption of N_2_ to the active sites, which requires the atoms containing not fully occupied orbitals to accept the lone-pair electrons from the *σ* orbital of N_2_. Therefore, transition metals would be ideal electron acceptors due to their half-occupied *d* orbitals, and they have the potential to be promising NRR electrocatalysts (Lan et al., 2013; Le et al., 2014; Han et al., 2019). Ling and co-workers established a computational model that anchored Mo atoms onto N-doped carbon (Mo_1_-N_1_C_2_) and studied its NRR catalytic performance using the first principle calculations (Ling et al., 2018b). Firstly, the bonding energy of N_2_ adsorbed on M_1_-N_1_C_2_ (M = Cu, Pd, Pt, and Mo) is investigated; only Mo_1_-N_1_C_2_ shows strong adsorption of N_2_ with the adsorption energy of −1.19 and −1.18 eV for side-on and end-on adsorption, respectively. In addition, the N≡N bond length has increased from 1.12 Å (isolated N_2_ molecule) to 1.18 Å (side-on) and 1.14 Å (end-on) (Figures 14A–C). Accordingly, Mo is selected as the potential electrocatalyst, and the possible reaction pathways are calculated. As shown in Figure 14 D, Mo_1_-N_1_C_2_ has a low overpotential of 0.24 V and it can catalyze the NRR through an enzymatic pathway. It is worth noting that the generated NH_3_ can be removed quickly from the Mo_1_-N_1_C_2_ catalyst with a free-energy uphill of only 0.47 eV, which is much lower than that of the previously reported catalysts.

In recent years, metal-free catalysts have also emerged as an important category of electrocatalysts for ammonia formation. Boron (B) has the potential to be used in NRR processes because of its Lewis acid-like characteristics and electron-deficient nature, which is different from other main group elements. The outer orbital of B undergoes hybridization to produce *sp* ^*2*^ orbitals, which can accept electrons from the N_2_ *σ*-bond and donate electrons from the filled *2p* orbitals to the antibonding π*-orbitals of N_2_. This finding has been supported by a number of recent investigations (Ling et al., 2018c; Yu et al., 2018; Mao et al., 2019c). For instance, Yu’s group reported that B-doped graphene could effectively catalyze the NRR with a high Faradic efficiency of 10.8% for NH_3_ production in aqueous solutions under ambient conditions at −0.5 V (*versus* RHE), with the electron redistribution at the active site constituting the reduction process (Yu et al., 2018). However, the theoretically calculated overpotential remains very high, so there is still a great need for more suitable substrates to sustain B atoms. In addition, the detailed reaction mechanism of the N_2_ activation and reduction for non-metallic catalysts is yet to be uncovered, suggesting that new methods of nitrogen capture and activation should be exploited to increase the efficiency of nitrogen fixation.

## 5 Conclusions and outlook

**FIGURE 15:**
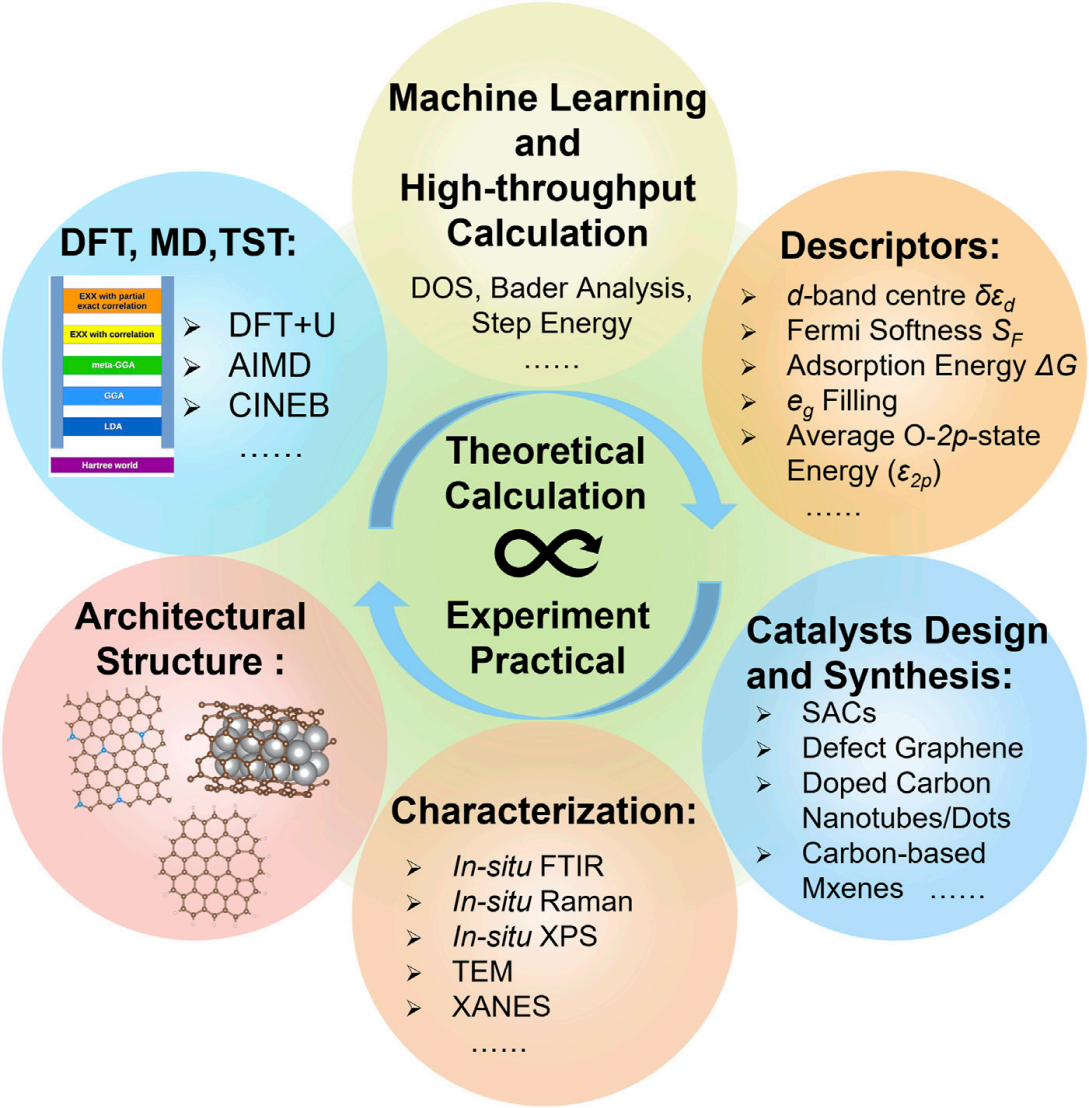
Schematic illustration showing the combination of theoretical calculations and experimental studies.

In summary, the development of modern DFT was briefly introduced, and the applications of theoretical calculations such as descriptors suited to material screening and mechanism studies in carbon-based heterogeneous catalysts were reviewed. Afterward, recent achievements of carbon-based electrocatalysts were presented. As shown in Figure 15, the development of supercomputer technology could improve the accuracy of the theoretical calculations. With the assistance of machine learning and high-throughput computing, the prediction and screening ability of computational simulation has gradually enhanced and become more closely integrated with experimental work. Besides, DFT calculations can simulate a more realistic reaction environment and material structures, which could improve the efficiency and accuracy of the mechanism explanation. Particularly, low-dimensional carbon-based materials have demonstrated tremendous potential in electrocatalysis due to their distinctive features, including 1) diverse and controllable structures as well as excellent environmental tolerance; 2) inherent substrate materials can be easily doped by heteroatoms; 3) various defects can serve as the active sites. However, similar to other kinds of excellent electrocatalysts, carbon-based electrocatalysts also face severe limitations in the rational design and practical application. More universal, accurate, and measurable descriptors should be developed, and additional research attention should be focused on the synthesis of catalysts. The following important issues and challenges deserve further investigation.

(1) Microkinetic modeling is a bridge that can connect quantum-chemical data with macroscopic behaviors in surface reactions. However, the existing theoretical approaches and functionals describing interface charge cannot meet the requirement of evaluating kinetics and reaction barriers of elementary steps under realistic reaction environments. For example, the widely used CHE model can only obtain the reaction-free energies, without the consideration of the non-electrochemical processes, proton-electron transfer, and the recombination of electrons and holes. More advancements in kinetic modeling are needed to fully uncover the relationship between coverage, potential dependency of activation energies, adsorbate-adsorbate interactions, and pH effects. For interfacial catalytic processes, multiscale modeling can facilitate the understanding of the transport effects. Alternatively, assisted by advanced sampling methods, such as slow-growth (SG), metadynamics, and umbrella sampling, AIMD can compensate for the shortcomings of the CHE models. Meanwhile, grand-canonic fixed-potential DFT calculations offer a feasible way to evaluate the catalytic performance at equilibrium states, which is the same as the experimental setup.
(2) The combination of multiple descriptors to effectively describe the relationship between structures and performance is indispensable. This is because it may be impossible to accurately predict the activity trends of complicated multi-phase catalysts only using one descriptor. Meanwhile, the simulation results of completed calculations, such as explicit SG-AIMD simulation, are highly dependent on the selection of collective variables. Importantly, machine learning, high-throughput calculations, and force-field approaches should be applied to uncover more general descriptors and advanced models, leading to higher efficient reaction mechanisms investigation and catalytic performance prediction.
(3) In addition to kinetics and reaction barriers, the stability of the catalysts plays a pivotal role in practical applications. Several commonly employed simulation methods are used to assess the stability of designed catalysts. For instance, retaining a stable structure during AIMD simulations is often interpreted as a sign of catalyst stability; nevertheless, the limited simulation duration reduces the reliability of the results. Another commonly used method, Pourbaix diagrams, cannot fully overcome the limitation of thermodynamic formation energy, even considering factors such as pH and applied potential. Hence, there is a pressing need to develop a dependable approach for evaluating the stability of electrocatalysts, which deserves more attention in research.
(4) Selectivity is also a critical parameter in assessing the catalytic performance of electrocatalysts. In simulations, the selectivity of electrocatalysts is often evaluated by comparing formation energies, adsorption energies of reactants, or energy barriers of potential determining steps (PDS). However, these evaluations are typically based on the CHE model and do not consider the real reaction environment, which substantially impacts the selectivity of catalysts. Consequently, it is still unclear how to effectively assess the selectivity, while microkinetic models that consider various environmental factors may prove to be a successful solution.
(5) Synthesis of electrocatalysts with abundant active sites is also crucial, for instance, the formation process of specific defect structures and the synthesis of SACs with specific density are yet to be improved. Theoretical scientists have been focusing on improving the activity of electrocatalysts. However, rational design and synthesis strategies for large-scale production of carbon-based electrocatalysts are also one of the stumbling blocks for practical applications. More attention should be focused on the synthesis of electrocatalysts with the targeted active site for a specific reaction.
(6) The scaling relationship between oxygen adsorbates in OER, ORR, and CO_2_RR has the possibility to develop efficient descriptors. However, it also restricts the lowest overpotential of electrocatalytic reactions, for example, the predicted lowest overpotential is (3.2–2.46) eV/2e ≈ 0.4 V for both ORR and OER. Therefore, how to break the scaling relationship is also a prospective research topic in the near future. One possible solution is stabilizing the key intermediates, and the other method is exploring new reaction mechanisms to avoid the formation of key intermediates.
